# Supplementary figures and images for: Elevated cytokines and chemokines in peripheral blood of patients with SARS-CoV-2 pneumonia treated with high-titer convalescent plasma
Source: PLoS Pathog. 2021 Oct 29;17(10):e1010025. doi: 10.1371/journal.ppat.1010025 (PMC8580259; doi:10.1371/journal.ppat.1010025)

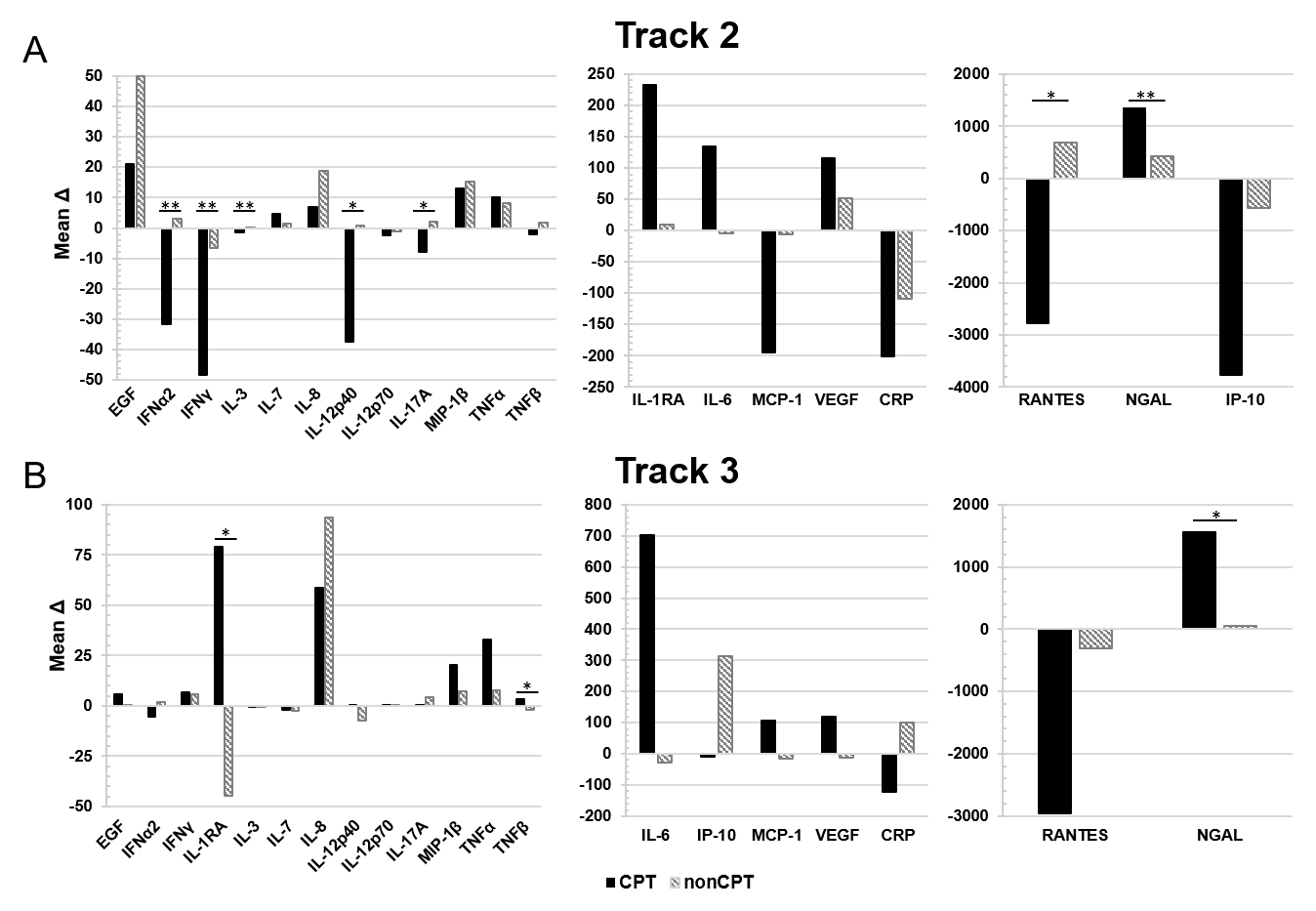

Supplement: S1 Fig — The mean change (Δ) in cytokine concentration between pre-infusion levels (Day 0) and Day 10 post-infusion for CPT patients and corresponding10-day period for nonCPT treated patients were calculated in patients categorized as Track 2 (A) and Track 3 (B). Statistically significant changes in mean values between those patient who received CPT (Track 2, n = 22; Track 3, n = 10) and those who did not (nonCPT; Track 2, n = 8; Track 3, n = 10) were evaluated via T-test. Significance is denoted by (*) for 0.01<p<0.05 or (**) for p<0.01. (TIF) [file ppat.1010025.s001.tif]
