# Supplementary material for: Elevated cytokines and chemokines in peripheral blood of patients with SARS-CoV-2 pneumonia treated with high-titer convalescent plasma
Source: PLoS Pathog. 2021 Oct 29;17(10):e1010025. doi: 10.1371/journal.ppat.1010025 (PMC8580259; doi:10.1371/journal.ppat.1010025)
Supplement: S1 Table — (DOCX) [file ppat.1010025.s002.docx]

| **S1 Table. Donor Plasma Luminex Analyses** | | | | | | | | |  | |  | |  | |  | |  | |  | |  | |  | |  |
| --- | --- | --- | --- | --- | --- | --- | --- | --- | --- | --- | --- | --- | --- | --- | --- | --- | --- | --- | --- | --- | --- | --- | --- | --- | --- |
| **Concentration (pg/ml) of Analytes with Elevation 0-19% of Donors ^δ^** | | | | | | |  | |  | |  | |  | |  | |  | |  | |  | |  | |  |
| **Donor** | **EGF** | **Eotaxin (CCL11)** | **G-CSF** | **GM-CSF** | **IFNγ** | **IL-1α** | | **IL-1β** | | **IL-1RA** | | **IL-2** | | **IL-3** | | **IL-4** | | **IL-5** | | **IL-7** | | **IL-8 (CXCL8)** | | **IL-10** | |
| Don01 | <3.2 | 108.85 | <4.8 | <2.56 | 19.30 | 3.63 | | 23.40 | | 3.30 | | <0.64 | | <1.28 | | <0.64 | | 1.06 | | <0.64 | | 0.87 | | <2.56 | |
| Don02 | 18.33 | 81.64 | <4.8 | <2.56 | 29.44 | 0.50 | | 7.83 | | 1.42 | | <0.64 | | <1.28 | | <0.64 | | 1.96 | | <0.64 | | 0.74 | | <2.56 | |
| Don03 | 21.67 | 86.32 | 81.35 | <2.56 | <1.28 | <4.8 | | <1.6 | | 0.53 | | <0.64 | | <1.28 | | 3.96 | | 1.22 | | <0.64 | | 0.67 | | 37.43 | |
| Don04 | <3.2 | 56.82 | <4.8 | <2.56 | 13.32 | <4.8 | | 3.20 | | 1.42 | | <0.64 | | <1.28 | | <0.64 | | 0.78 | | <0.64 | | 0.11 | | <2.56 | |
| Don05 | 0.07 | 73.43 | <4.8 | <2.56 | 16.29 | 2.11 | | 20.92 | | 3.88 | | <0.64 | | <1.28 | | <0.64 | | 5.03 | | 2.62 | | 0.64 | | <2.56 | |
| Don06 | 14.01 | 65.34 | <4.8 | <2.56 | 4.49 | 0.63 | | <1.6 | | 3.02 | | <0.64 | | <1.28 | | <0.64 | | 2.58 | | <0.64 | | 0.83 | | <2.56 | |
| Don07 | <3.2 | 26.95 | <4.8 | <2.56 | 20.76 | <4.8 | | 1.04 | | 1.57 | | <0.64 | | <1.28 | | <0.64 | | 1.32 | | <0.64 | | 0.25 | | <2.56 | |
| Don08 | 40.24 | 80.60 | 191.64 | <2.56 | 20.03 | 6.12 | | 23.02 | | 5.86 | | 0.14 | | <1.28 | | 8.95 | | 8.03 | | <0.64 | | 1.44 | | 82.37 | |
| Don09 | 31.12 | 92.04 | 93.34 | <2.56 | 5.42 | 0.51 | | 1.75 | | 1.64 | | <0.64 | | <1.28 | | 3.86 | | 3.10 | | <0.64 | | 1.53 | | 56.34 | |
| Don10 * | 10.62 | 30.22 | <4.8 | <2.56 | 37.31 | 51.39 | | 6.82 | | 3.91 | | <0.64 | | <1.28 | | <0.64 | | 4.08 | | <0.64 | | 0.95 | | <2.56 | |
| Don11 | 10.62 | 30.22 | <4.8 | <2.56 | 37.31 | 51.39 | | 6.82 | | 3.91 | | <0.64 | | <1.28 | | <0.64 | | 4.08 | | <0.64 | | 0.95 | | <2.56 | |
| Don12 | 24.21 | 75.80 | <4.8 | <2.56 | 37.75 | 0.63 | | 10.46 | | 2.01 | | <0.64 | | <1.28 | | <0.64 | | 6.03 | | <0.64 | | 0.73 | | <2.56 | |
| Don13 | <3.2 | 106.65 | <4.8 | <2.56 | 2.14 | 0.62 | | <1.6 | | 0.68 | | <0.64 | | <1.28 | | <0.64 | | 2.43 | | <0.64 | | 0.81 | | <2.56 | |
| Don14 | 25.26 | 84.58 | 119.98 | <2.56 | 14.82 | 3.63 | | 15.68 | | 4.87 | | <0.64 | | <1.28 | | 2.61 | | 4.27 | | <0.64 | | 0.88 | | 29.91 | |
| Don15 | <3.2 | 83.76 | <4.8 | <2.56 | 7.10 | <4.8 | | <1.6 | | 0.75 | | <0.64 | | <1.28 | | <0.64 | | 2.27 | | <0.64 | | 0.81 | | <2.56 | |
| Don16 | 10.62 | 72.56 | <4.8 | <2.56 | 22.97 | 0.62 | | 2.99 | | 1.13 | | 0.23 | | <1.28 | | <0.64 | | 1.43 | | <0.64 | | 0.71 | | <2.56 | |
| Don17 | 9.37 | 38.64 | <4.8 | <2.56 | 10.25 | 0.63 | | 0.30 | | 1.64 | | <0.64 | | <1.28 | | <0.64 | | 2.99 | | <0.64 | | 0.50 | | <2.56 | |
| Don18 | 18.12 | 58.47 | 16.55 | <2.56 | 10.17 | 0.16 | | 4.44 | | 2.01 | | <0.64 | | <1.28 | | 0.81 | | 1.85 | | <0.64 | | 1.76 | | 3.68 | |
| Don19 | 15.94 | 38.53 | <4.8 | <2.56 | 37.89 | 5.72 | | 20.94 | | 5.58 | | 0.69 | | 0.30 | | 0.27 | | 5.23 | | <0.64 | | 1.69 | | <2.56 | |
| Don21 | <3.2 | 114.86 | <4.8 | <2.56 | 5.42 | 0.86 | | 0.30 | | 0.90 | | <0.64 | | <1.28 | | <0.64 | | 2.99 | | <0.64 | | 0.25 | | <2.56 | |
| Don22 * | <3.2 | 114.86 | <4.8 | <2.56 | 5.42 | 0.86 | | 0.30 | | 0.90 | | <0.64 | | <1.28 | | <0.64 | | 2.99 | | <0.64 | | 0.25 | | <2.56 | |
| Don23 | <3.2 | 32.89 | 8.51 | <2.56 | 7.10 | 1.35 | | 1.75 | | 1.86 | | <0.64 | | <1.28 | | 0.17 | | 1.22 | | <0.64 | | 0.64 | | <2.56 | |
| Don24 | <3.2 | 69.79 | <4.8 | <2.56 | 3.82 | 4.8 | | <1.6 | | 0.83 | | <0.64 | | <1.28 | | <0.64 | | 2.17 | | 2.28 | | 0.76 | | <2.56 | |
| Don25 | 20.05 | 55.18 | 65.63 | <2.56 | 10.25 | 1.61 | | 8.95 | | 2.44 | | <0.64 | | <1.28 | | 2.50 | | 3.35 | | <0.64 | | 0.90 | | 24.65 | |
| Don26 | 5.30 | 127.46 | <4.8 | <2.56 | 17.81 | 5.85 | | 32.10 | | 7.83 | | <0.64 | | <1.28 | | <0.64 | | 10.02 | | <0.64 | | 1.27 | | <2.56 | |
| Don27 | 3.2 | 46.77 | <4.8 | <2.56 | 7.73 | 4.8 | | 12.92 | | 1.71 | | <0.64 | | <1.28 | | <0.64 | | 1.32 | | <0.64 | | 1.02 | | <2.56 | |
| Don29 | 12.66 | 29.12 | <4.8 | <2.56 | 35.05 | 2.37 | | 15.17 | | 3.16 | | 0.57 | | <1.28 | | <0.64 | | 3.15 | | <0.64 | | 0.85 | | <2.56 | |
| Don33 ** | 0.74 | 30.07 | <4.8 | <2.56 | 11.77 | 1.35 | | 7.36 | | 2.15 | | <0.64 | | <1.28 | | <0.64 | | 2.17 | | <0.64 | | 0.53 | | <2.56 | |
| Don34 | <3.2 | 105.33 | <4.8 | <2.56 | <1.28 | 0.86 | | <1.6 | | 0.83 | | <0.64 | | <1.28 | | <0.64 | | 1.00 | | <0.64 | | 1.02 | | <2.56 | |
| Don35 | <3.2 | 99.32 | <4.8 | <2.56 | 2.14 | <4.8 | | 0.30 | | 1.71 | | <0.64 | | <1.28 | | <0.64 | | 1.96 | | <0.64 | | 1.01 | | <2.56 | |
| Don36 | <3.2 | 90.73 | <4.8 | <2.56 | <1.28 | <4.8 | | <1.6 | | 0.22 | | <0.64 | | <1.28 | | <0.64 | | 1.32 | | <0.64 | | 0.25 | | <2.56 | |
| Don37 | <3.2 | 108.71 | <4.8 | <2.56 | 8.69 | 0.39 | | 42.36 | | 1.13 | | <0.64 | | <1.28 | | <0.64 | | 1.00 | | <0.64 | | 1.04 | | <2.56 | |
| Don38 | <3.2 | 20.00 | <4.8 | <2.56 | 3.82 | 1.48 | | <1.6 | | 1.71 | | <0.64 | | <1.28 | | <0.64 | | 1.27 | | <0.64 | | 0.53 | | <2.56 | |
| Don39 | 0.74 | 30.07 | <4.8 | <2.56 | 11.77 | 1.35 | | 7.36 | | 2.15 | | <0.64 | | <1.28 | | <0.64 | | 2.17 | | <0.64 | | 0.53 | | <2.56 | |
| Don40 | 1.95 | 119.90 | 22.71 | <2.56 | <1.28 | <4.8 | | <1.6 | | 0.37 | | <0.64 | | <1.28 | | 0.63 | | 1.64 | | <0.64 | | 0.18 | | 7.62 | |
| **Mean** | 9.70 | 71.04 | 20.84 | 2.56 | 13.79 | 5.42 | | 8.37 | | 2.26 | | 0.61 | | 1.25 | | 1.15 | | 2.84 | | 0.74 | | 0.80 | | 8.96 | |
| **SD** | 9.82 | 32.06 | 40.96 | 0.00 | 11.85 | 11.65 | | 10.13 | | 1.73 | | 0.11 | | 0.17 | | 1.62 | | 2.04 | | 0.43 | | 0.40 | | 17.41 | |
| **% Elevated** | 2.86 | 0.00 | 2.86 | 0.00 | 17.14 | 5.71 | | 5.71 | | 0.00 | | 2.86 | | 0.00 | | 0.00 | | 0.00 | | 5.71 | | 0.00 | | 2.86 | |
| Control Mean +2xSD | 33.40 | 248.53 | 137.32 | 2.56 | 25.90 | 11.05 | | 28.09 | | 37.00 | | 0.64 | | 1.28 | | 9.29 | | 16.93 | | 0.64 | | 2.37 | | 56.39 | |

| **S1 Table. Donor Plasma Luminex Analyses (continued)** | | | | | | | |  |  |  |  |  |  |  |  |  |
| --- | --- | --- | --- | --- | --- | --- | --- | --- | --- | --- | --- | --- | --- | --- | --- | --- |
| **Concentration (pg/ml) of Analytes with Elevation 0-19% of Donors ^δ^** | | | | | | | |  |  |  |  |  |  |  |  |  |
| **Donor** | **IL-12p40** | **IL-12p70** | **IL-13** | **IL-15** | **IL-17A** | **IP-10 (CXCL10)** | **MCP-1 (CCL2)** | **MIP-1α (CCL3)** | **MIP-1β (CCL4)** | **RANTES (CCL5)** | **TNFα** | **TNFβ** | **VEGF** | **MBL (ng/ml)** | **NGAL (ng/ml)** | **SP-D** |
| Don01 | 16.03 | <3.2 | <6.4 | 10.56 | 17.14 | 45.55 | 293.67 | <3.2 | 11.94 | 890.49 | 7.08 | <1.6 | <2.56 | 8974.8 | 75.66 | 66.53 |
| Don02 | 16.00 | 4.06 | 38.65 | 8.33 | 4.55 | 42.94 | 203.57 | 6.98 | 23.75 | 2038.48 | 17.34 | <1.6 | <2.56 | 328.0 | 47.11 | 19.19 |
| Don03 | 20.88 | <3.2 | <6.4 | 4.27 | <1.28 | 122.87 | 298.96 | <3.2 | 13.52 | 706.87 | 6.25 | <1.6 | 17.45 | 540.4 | 71.31 | 2.39 |
| Don04 | <6.4 | 2.39 | 13.72 | 4.27 | 7.33 | 270.75 | 124.89 | <3.2 | 10.59 | 987.10 | 2.25 | 3.01 | <2.56 | 9770.2 | 75.57 | 7.70 |
| Don05 | 34.01 | <3.2 | 74.99 | 25.26 | 4.45 | 66.34 | 261.47 | 4.04 | 13.97 | 3456.46 | 21.02 | 28.14 | <2.56 | 52.7 | 72.15 | 4.28 |
| Don06 | 32.98 | <3.2 | <6.4 | 4.86 | 2.29 | 92.75 | 282.04 | <3.2 | 25.08 | 2900.12 | 12.54 | <1.6 | 2.74 | 1109.3 | 78.45 | 21.93 |
| Don07 | 29.34 | <3.2 | <6.4 | 5.44 | <1.28 | 54.40 | 217.78 | <3.2 | 21.66 | 209.88 | 9.28 | <1.6 | <2.56 | 633.8 | 53.67 | 42.37 |
| Don08 | 27.25 | 1.45 | 16.21 | 29.89 | 14.70 | 172.52 | 467.00 | 27.88 | 25.61 | 2186.10 | 15.48 | <1.6 | 48.98 | 118.6 | 52.88 | 83.18 |
| Don09 | 19.27 | <3.2 | <6.4 | 7.84 | 2.53 | 119.32 | 496.09 | <3.2 | 46.31 | 1224.57 | 17.60 | <1.6 | 29.67 | 2396.6 | 56.12 | 25.78 |
| Don10 * | 32.88 | 5.54 | 12.44 | 12.47 | 4.17 | 53.93 | 232.49 | <3.2 | 24.13 | 209.87 | 23.63 | <1.6 | 4.44 | 604.6 | 51.55 | 37.79 |
| Don11 | 32.88 | 5.54 | 12.44 | 12.47 | 4.17 | 53.93 | 232.49 | <3.2 | 24.13 | 209.87 | 23.63 | <1.6 | 4.44 | 604.6 | 51.55 | 37.79 |
| Don12 | 11.60 | 15.64 | 55.91 | 9.92 | 1.59 | 69.32 | 267.66 | <3.2 | 17.22 | 2308.13 | 28.56 | 27.51 | <2.56 | 6027.2 | 51.16 | 55.55 |
| Don13 | 26.20 | 0.29 | 29.10 | 5.81 | <1.28 | 99.79 | 333.84 | <3.2 | 25.04 | 1236.73 | 13.88 | 9.27 | <2.56 | 2323.9 | 76.47 | 10.04 |
| Don14 | 29.86 | 2.39 | 12.00 | 17.76 | 10.56 | 69.89 | 367.26 | 18.85 | 30.50 | 2204.52 | 16.54 | 1.05 | 10.19 | 3843.9 | 45.66 | 74.95 |
| Don15 | 39.65 | <3.2 | <6.4 | 4.87 | 0.88 | 126.69 | 327.09 | <3.2 | 22.42 | 524.23 | 2.84 | <1.6 | 0.25 | 1457.1 | 53.45 | 9.68 |
| Don16 | 25.67 | 2.39 | 47.07 | 6.70 | 2.53 | 86.28 | 331.90 | 51.42 | 14.94 | 1281.24 | 22.06 | 6.88 | <2.56 | 764.0 | 63.69 | 14.00 |
| Don17 | 24.09 | 1.59 | 6.58 | 5.06 | 5.00 | 75.40 | 174.31 | <3.2 | 17.38 | 2875.98 | 8.46 | <1.6 | <2.56 | 1847.1 | 46.46 | 4.27 |
| Don18 | 34.01 | 2.24 | <6.4 | 8.72 | 4.17 | 112.12 | 398.95 | <3.2 | 28.24 | 4337.30 | 19.18 | <1.6 | 7.08 | 3449.2 | 64.21 | 9.48 |
| Don19 | 44.22 | 8.97 | 57.36 | 13.70 | 16.54 | 48.06 | 145.55 | 27.88 | 30.50 | 3055.28 | 22.05 | 4.64 | 4.73 | 385.4 | 70.76 | 7.75 |
| Don21 | 28.82 | 1.13 | <6.4 | 10.09 | <1.28 | 85.48 | 304.82 | <3.2 | 20.51 | 1402.39 | 13.88 | <1.6 | <2.56 | 3969.1 | 64.33 | 35.89 |
| Don22 * | 28.82 | 1.13 | <6.4 | 10.09 | <1.28 | 85.48 | 304.82 | <3.2 | 20.51 | 1402.39 | 13.88 | <1.6 | <2.56 | 3969.1 | 64.33 | 35.89 |
| Don23 | 18.20 | 0.64 | <6.4 | 8.02 | 2.07 | 125.68 | 305.46 | <3.2 | 21.66 | 2517.01 | 17.07 | <1.6 | 5.32 | 121.3 | 56.90 | 20.71 |
| Don24 | 30.38 | 0.97 | <6.4 | 4.21 | 0.88 | 46.34 | 224.78 | <3.2 | 13.85 | 3658.66 | 5.41 | <1.6 | <2.56 | 235.2 | 63.80 | 7.23 |
| Don25 | 44.19 | 3.16 | 12.00 | 10.90 | 3.23 | 89.00 | 276.84 | <3.2 | 32.58 | 3595.89 | 13.07 | <1.6 | 17.66 | 489.5 | 71.41 | 22.95 |
| Don26 | 28.82 | 0.64 | 31.04 | 37.75 | 8.80 | 47.66 | 225.36 | 58.43 | 32.30 | 1717.27 | 13.33 | 1.72 | <2.56 | 4303.4 | 41.93 | 58.54 |
| Don27 | 17.08 | <3.2 | 42.74 | 6.89 | 0.88 | 99.75 | 193.24 | <3.2 | 17.22 | 1297.05 | 6.25 | <1.6 | <2.56 | 264.1 | 48.94 | 15.86 |
| Don29 | 28.82 | 5.83 | 313.93 | 9.74 | 9.52 | 40.95 | 147.35 | 14.91 | 26.26 | 3321.48 | 46.37 | 60.22 | <2.56 | 3127.8 | 41.35 | 2.22 |
| Don33 ** | 24.60 | 1.93 | <6.4 | 5.25 | 5.00 | 42.38 | 131.00 | <3.2 | 19.63 | 2837.67 | 10.65 | <1.6 | <2.56 | 12400.4 | 52.82 | 10.24 |
| Don34 | 4.67 | <3.2 | <6.4 | 7.12 | 1.59 | 100.38 | 267.93 | <3.2 | 39.55 | 1419.26 | 7.08 | <1.6 | <2.56 | 101.0 | 53.88 | 4.56 |
| Don35 | 19.82 | 0.64 | <6.4 | 6.18 | <1.28 | 77.14 | 220.16 | <3.2 | 17.22 | 1125.79 | 9.00 | <1.6 | <2.56 | 2910.2 | 83.58 | 7.43 |
| Don36 | 13.80 | <3.2 | <6.4 | 3.86 | <1.28 | 47.46 | 310.98 | <3.2 | 25.46 | 855.33 | 6.4 | <1.6 | <2.56 | 1074.2 | 74.49 | 5.93 |
| Don37 | 20.36 | <3.2 | <6.4 | 8.01 | <1.28 | 130.09 | 229.47 | <3.2 | 25.61 | 1469.59 | 86.92 | <1.6 | <2.56 | 1843.8 | 50.93 | 8.58 |
| Don38 | 24.62 | 0.47 | <6.4 | 9.24 | 2.29 | 52.25 | 242.82 | <3.2 | 22.40 | 2070.08 | 14.95 | <1.6 | <2.56 | 5552.9 | 73.94 | 35.16 |
| Don39 | 24.60 | 1.93 | <6.4 | 5.25 | 5.00 | 42.38 | 131.00 | <3.2 | 19.63 | 2837.67 | 10.65 | <1.6 | <2.56 | 12400.4 | 52.82 | 10.24 |
| Don40 | 31.94 | 0.29 | <6.4 | 2.35 | <1.28 | 47.75 | 536.34 | <3.2 | 18.31 | 1143.51 | 6.53 | <1.6 | 8.28 | 511.7 | 67.36 | 4.38 |
| **Mean** | 25.51 | 3.04 | 25.65 | 9.80 | 4.38 | 84.09 | 271.70 | 8.48 | 22.85 | 1871.84 | 16.32 | 5.26 | 6.22 | 2814.44 | 60.59 | 23.44 |
| **SD** | 9.25 | 2.84 | 53.35 | 7.48 | 4.45 | 46.18 | 98.40 | 13.30 | 7.60 | 1094.50 | 14.94 | 11.43 | 9.41 | 3403.35 | 11.71 | 22.06 |
| **% Elevated** | 0.00 | 17.1 | 14.29 | 2.86 | 5.71 | 5.71 | 14.29 | 0.00 | 11.43 | 0.00 | 5.71 | 11.43 | 0.00 | 11.43 | 0.00 | 11.43 |
| Control Mean + 2xSD | 46.10 | 3.68 | 44.99 | 32.43 | 14.88 | 144.98 | 338.62 | 73.36 | 31.57 | 4816.80 | 31.96 | 8.28 | 54.93 | 6380.78 | 380.66 | 106.54 |

^δ^ Concentration values in gray highlight are considered elevated above the normal control mean +
