# Supplementary material for: Elevated cytokines and chemokines in peripheral blood of patients with SARS-CoV-2 pneumonia treated with high-titer convalescent plasma
Source: PLoS Pathog. 2021 Oct 29;17(10):e1010025. doi: 10.1371/journal.ppat.1010025 (PMC8580259; doi:10.1371/journal.ppat.1010025)
Supplement: S2 Table — (DOCX) [file ppat.1010025.s003.docx]

| **S2 Table. Recipient Plasma Luminex Analyses**  **Pre-Infusion Concentration (pg/ml) of Analytes with Elevation 0-19% of Recipients ^δ^** | | | | | | | | |  | |  | |  | |  | |  | |  |  |  |
| --- | --- | --- | --- | --- | --- | --- | --- | --- | --- | --- | --- | --- | --- | --- | --- | --- | --- | --- | --- | --- | --- |
| **Recipient** | **Eotaxin (CCL11)** | **G-CSF** | **GM-CSF** | **IL-1α** | **IL-1β** | **IL-2** | **IL-4** | **IL-5** | | **IL-10** | | **IL-13** | | **IL-15** | | **MIP-1a (CCL3)** | | **MBL ng/ml** | | **PCT ng/ml** | **SP-D** |
| REC01 | 29.06 | 6.90 | <2.56 | 4.07 | 24.59 | <0.64 | 0.22 | 4.69 | | 25.76 | | 28.90 | | 9.31 | | 28.07 | | 1932.64 | | 37.05 | 0.23 |
| REC02 | 113.38 | <4.8 | <2.56 | <4.8 | 2.28 | <0.64 | <0.64 | 2.18 | | <2.56 | | <6.4 | | 3.83 | | <3.2 | | 265.56 | | 26.63 | 13.96 |
| REC03 | 158.57 | 63.27 | <2.56 | 8.25 | 19.42 | <0.64 | 1.16 | 7.62 | | 4.68 | | 56.91 | | 13.68 | | 46.66 | | 1152.44 | | 40.02 | 28.62 |
| REC04 | 90.12 | <4.8 | <2.56 | 3.01 | 9.22 | <0.64 | 0.82 | 2.96 | | <2.56 | | 15.84 | | 6.70 | | 6.60 | | 267.60 | | 127.04 | 2.37 |
| REC05 | 80.44 | 34.44 | 61.57 | 2.70 | 19.86 | 0.92 | <0.64 | 5.42 | | 15.85 | | 16.42 | | 19.14 | | 72.64 | | 2668.19 | | 94.31 | 30.97 |
| REC06 | 173.14 | 50.54 | <2.56 | 14.84 | 78.42 | 31.03 | 1.68 | 7.38 | | 3.70 | | 44.88 | | 18.15 | | 71.23 | | 1541.16 | | 42.25 | 1.21 |
| REC07 | 75.00 | <4.8 | <2.56 | 1.19 | 3.25 | <0.64 | <0.64 | 1.56 | | 4.92 | | <6.4 | | 16.68 | | <3.2 | | 1392.59 | | 9.53 | 22.97 |
| REC08 | 23.08 | 30.87 | <2.56 | 5.79 | 15.02 | 1.15 | 0.32 | 4.60 | | 18.63 | | 26.41 | | 10.35 | | 32.86 | | 8142.99 | | 122.57 | 37.51 |
| REC09 | 136.78 | 19.48 | <2.56 | 7.80 | 19.35 | <0.64 | 0.59 | 6.03 | | 16.81 | | 41.60 | | 15.64 | | 37.88 | | 6191.47 | | 10.27 | 9.72 |
| REC10 | 106.98 | 334.19 | <2.56 | <4.8 | <1.6 | <0.64 | <0.64 | 5.18 | | 21.89 | | <6.4 | | 12.36 | | 19.84 | | 233.00 | | 143.40 | 56.47 |
| REC11 | 55.61 | 13803.18 | <2.56 | <4.8 | 1.54 | <0.64 | <0.64 | 11.86 | | 2.84 | | <6.4 | | 33.08 | | <3.2 | | 1332.02 | | 124.06 | 56.08 |
| REC12 | 33.87 | <4.8 | <2.56 | 1.21 | 3.73 | <0.64 | 1.34 | 1.56 | | 26.36 | | 7.15 | | 9.04 | | <3.2 | | 108.68 | | 43.74 | 6.25 |
| REC13 | 71.86 | 143.94 | <2.56 | 17.03 | 24.76 | 2.46 | 1.15 | 7.30 | | 47.34 | | 57.94 | | 26.77 | | 64.60 | | 4029.51 | | 34.81 | 16.23 |
| REC14 | 44.19 | 9.22 | <2.56 | 1.61 | 12.40 | <0.64 | <0.64 | 1.81 | | <2.56 | | 9.16 | | 7.93 | | 13.27 | | 1016.78 | | 6.55 | 33.69 |
| REC15 | 41.02 | 7.57 | <2.56 | 7.33 | 17.02 | 0.53 | 0.70 | 5.42 | | 24.19 | | 40.77 | | 11.81 | | 36.10 | | 1364.25 | | 71.26 | 57.08 |
| REC16 | 110.81 | <4.8 | <2.56 | 3.20 | 15.50 | <0.64 | 2.35 | 1.52 | | <2.56 | | 27.13 | | 5.39 | | 12.30 | | 8223.31 | | 61.59 | 10.25 |
| REC17 | 74.95 | <4.8 | <2.56 | 2.23 | 10.17 | <0.64 | 0.34 | 7.01 | | 19.84 | | 22.67 | | 13.13 | | 23.21 | | 5314.38 | | 58.61 | 35.81 |
| REC18 | 91.61 | <4.8 | <2.56 | <4.8 | <1.6 | <0.64 | <0.64 | 1.47 | | 18.26 | | <6.4 | | 6.08 | | <3.2 | | 9818.21 | | 9.53 | 13.67 |
| REC19 | 101.94 | <4.8 | <2.56 | <4.8 | <1.6 | <0.64 | <0.64 | 1.18 | | 10.15 | | <6.4 | | 6.56 | | <3.2 | | 4982.65 | | 179.10 | 43.62 |
| REC21 | 18.43 | <4.8 | <2.56 | 1.60 | 7.39 | <0.64 | 0.34 | 1.39 | | 14.39 | | 10.26 | | 11.95 | | 15.81 | | 1094.86 | | 109.93 | 37.12 |
| REC22 | 142.22 | <4.8 | <2.56 | 2.03 | 4.66 | <0.64 | 0.32 | 0.88 | | 6.87 | | <6.4 | | 11.87 | | <3.2 | | 1230.51 | | 0.00 | 30.75 |
| REC23 | 176.81 | 18.54 | <2.56 | 7.10 | 44.31 | 0.92 | 0.85 | 4.69 | | 11.00 | | 28.80 | | 15.64 | | 40.25 | | 2486.36 | | 0.00 | 60.32 |
| REC24 | 90.56 | 38.62 | <2.56 | 10.46 | 22.64 | 1.01 | 1.15 | 9.58 | | 21.65 | | 65.32 | | 14.10 | | 48.76 | | 6104.44 | | 156.04 | 0.44 |
| REC25 | 58.76 | 21.79 | <2.56 | 9.06 | 33.90 | 2.37 | 2.59 | 9.95 | | 19.23 | | 82.27 | | 19.35 | | 68.57 | | 116.07 | | 197.69 | 40.11 |
| REC26 | 101.76 | <4.8 | <2.56 | <4.8 | 0.77 | <0.64 | 0.12 | 7.38 | | <2.56 | | <6.4 | | 7.18 | | <3.2 | | 4590.27 | | 101.75 | 45.35 |
| REC27 | 73.18 | <4.8 | <2.56 | 1.81 | <1.6 | <0.64 | <0.64 | 1.47 | | <2.56 | | <6.4 | | 4.98 | | <3.2 | | 1526.48 | | 0.00 | 31.05 |
| REC29 | 46.07 | 44.60 | <2.56 | 2.66 | 12.85 | <0.64 | 4.82 | 12.35 | | 41.38 | | 34.91 | | 16.05 | | 17.98 | | 841.88 | | 229.67 | 63.71 |
| REC33 | 47.30 | <4.8 | <2.56 | 0.79 | 3.22 | <0.64 | <0.64 | 1.64 | | 18.99 | | <6.4 | | 11.60 | | <3.2 | | 1156.34 | | 89.85 | 9.90 |
| REC34 | 17.95 | <4.8 | <2.56 | 0.40 | 4.20 | <0.64 | <0.64 | 0.67 | | <2.56 | | <6.4 | | 13.75 | | 41.20 | | 6169.69 | | 56.38 | 51.37 |
| REC35 | 60.88 | <4.8 | <2.56 | 0.12 | 9.72 | <0.64 | 0.53 | 4.85 | | 16.21 | | 18.09 | | 8.83 | | 19.26 | | 65.86 | | 120.34 | 13.64 |
| REC36 | 25.34 | <4.8 | <2.56 | 29.56 | 6.07 | <0.64 | 1.75 | 3.80 | | 4.68 | | 90.45 | | 16.89 | | <3.2 | | 319.13 | | 450.55 | 48.95 |
| REC37 | 150.88 | <4.8 | <2.56 | 0.30 | <1.6 | <0.64 | <0.64 | 0.97 | | <2.56 | | <6.4 | | 8.42 | | 33.89 | | 133.93 | | 34.81 | 10.45 |
| REC38 | 80.83 | <4.8 | <2.56 | <4.8 | 3.01 | <0.64 | 0.12 | 1.89 | | 14.88 | | 5.97 | | 8.83 | | <3.2 | | 4166.14 | | 220.74 | 0.96 |
| REC39 | 83.52 | <4.8 | <2.56 | 2.66 | 6.53 | <0.64 | <0.64 | 0.88 | | <2.56 | | <6.4 | | 9.80 | | <3.2 | | 730.23 | | 109.19 | 10.82 |
| REC40 | 66.81 | 37.75 | <2.56 | 4.07 | 16.59 | 0.60 | 0.74 | 5.46 | | 3.33 | | 39.09 | | 14.80 | | 40.05 | | 2418.74 | | 57.87 | 19.17 |
| **Mean** | 81.53 | 421.60 | 4.25 | 5.33 | 13.15 | 1.65 | 0.92 | 4.42 | | 13.05 | | 24.40 | | 12.56 | | 23.79 | | 2660.81 | | 90.78 | 27.17 |
| **SD** | 43.97 | 2329.19 | 9.97 | 5.70 | 15.29 | 5.13 | 0.88 | 3.28 | | 11.30 | | 23.26 | | 6.07 | | 22.38 | | 2684.90 | | 89.68 | 19.74 |
| **% Elevated** | 0.00 | 8.57 | 2.86 | 8.57 | 8.57 | 17.10 | 0.00 | 0.00 | | 0.00 | | 14.29 | | 2.86 | | 0.00 | | 8.57 | | 0.00 | 14.29 |
| Control Mean +2xSD | 248.53 | 137.32 | 2.56 | 11.05 | 28.09 | 0.64 | 9.29 | 16.93 | | 56.39 | | 44.99 | | 32.43 | | 73.36 | | 6380.78 | | 438.08 | 106.54 |

^δ^ Concentration values in gray highlight are considered elevated above the normal control mean + 2xSD
