# Supplementary material for: Elevated cytokines and chemokines in peripheral blood of patients with SARS-CoV-2 pneumonia treated with high-titer convalescent plasma
Source: PLoS Pathog. 2021 Oct 29;17(10):e1010025. doi: 10.1371/journal.ppat.1010025 (PMC8580259; doi:10.1371/journal.ppat.1010025)
Supplement: S3 Table — (DOCX) [file ppat.1010025.s004.docx]

| **S3 Table. Recipient Plasma Luminex Analyses** | | | | | | | | |  |  | |  | |  | |  | |  |  | |  |  |  |
| --- | --- | --- | --- | --- | --- | --- | --- | --- | --- | --- | --- | --- | --- | --- | --- | --- | --- | --- | --- | --- | --- | --- | --- |
| **Day 3 Concentration (pg/ml) of Analytes with Elevation 0-19% of Recipients ^δ^** | | | | | | | | |  |  |  |  |  |  |  |  |  |  |  |  |  |  |  |
| **Recipient** | **Eotaxin (CCL11)** | **G-CSF** | **GM-CSF** | **IL-1α** | **IL-1β** | **IL-2** | **IL-4** | **IL-5** | | | **IL-10** | | **IL-13** | | **IL-15** | | **MIP-1α (CCL3)** | **MBL ng/ml** | | **PCT ng/ml** | | | **SP-D** |
| REC01 | 31.98 | <4.8 | <2.56 | 2.11 | 29.31 | 0.09 | 0.30 | 6.03 | | | 7.03 | | 27.34 | | 9.51 | | 25.61 | 1900.15 | | 219.09 | | | 10.44 |
| REC02 | 50.13 | <4.8 | <2.56 | <4.8 | 3.10 | <0.64 | <0.64 | 1.75 | | | <2.56 | | <6.4 | | 3.34 | | <3.2 | 283.86 | | 1529.82 | | | 3.58 |
| REC03 | 142.77 | <4.8 | <2.56 | 2.20 | 7.41 | <0.64 | 0.58 | 2.63 | | | <2.56 | | 2.39 | | 4.29 | | 19.68 | 1028.54 | | 234.12 | | | 5.32 |
| REC04 | 123.70 | 7.95 | <2.56 | 0.55 | 1.60 | <0.64 | <0.64 | 37.69 | | | <2.56 | | <6.4 | | 5.36 | | <3.2 | 629.90 | | 285.23 | | | 2.81 |
| REC05 | 154.54 | 12.35 | <2.56 | 3.98 | 14.26 | <0.64 | 0.35 | 4.64 | | | 6.15 | | 23.85 | | 14.10 | | 27.88 | 3486.21 | | 183.02 | | | 19.01 |
| REC06 | 235.55 | 40.81 | <2.56 | 13.37 | 52.17 | 22.5 | 0.62 | 7.34 | | | 2.32 | | 25.39 | | 14.70 | | 52.27 | 2639.15 | | 26.69 | | | 0.48 |
| REC07 | 149.23 | <4.8 | <2.56 | 0.64 | 2.88 | <0.64 | <0.64 | 1.53 | | | <2.56 | | <6.4 | | 10.24 | | <3.2 | 1273.07 | | 155.96 | | | 8.36 |
| REC08 | 70.26 | 17.92 | <2.56 | 2.40 | 6.96 | 0.13 | 0.36 | 4.68 | | | 10.00 | | <6.4 | | 18.08 | | 43.41 | 330.19 | | 276.21 | | | 29.1 |
| REC09 | 99.05 | 14.30 | <2.56 | 2.01 | 6.75 | <0.64 | <0.64 | 10.35 | | | 10.40 | | 9.42 | | 12.50 | | 3.14 | 6470.34 | | 125.9 | | | 9.81 |
| REC10 | 80.63 | 2553.84 | <2.56 | 0.82 | 4.25 | 0.28 | <0.64 | 3.33 | | | 9.67 | | <6.4 | | 15.89 | | 14.27 | 408.71 | | 23.68 | | | 35.58 |
| REC11 | 39.35 | 15973.2 | 3.32 | 2.39 | <1.6 | <0.64 | <0.64 | 1.84 | | | <2.56 | | 22.43 | | 43.60 | | 50.32 | 691.63 | | 438.55 | | | 34.58 |
| REC12 | 34.23 | 6.08 | <2.56 | 3.99 | 12.29 | 0.88 | 0.62 | 8.74 | | | 41.52 | | 21.42 | | 11.17 | | 32.02 | 7991.07 | | 225.1 | | | 28.59 |
| REC13 | 119.96 | 142.88 | 4.96 | 25.36 | 46.95 | 3.90 | 3.02 | 14.76 | | | 25.73 | | 117.62 | | 28.70 | | 86.31 | 4040.48 | | 161.97 | | | 17.49 |
| REC14 | 90.81 | <4.8 | <2.56 | 1.26 | 6.97 | <0.64 | 1.48 | 1.40 | | | <2.56 | | 15.12 | | 5.76 | | 3.46 | 788.91 | | 56.75 | | | 33.61 |
| REC15 | 80.25 | <4.8 | <2.56 | <4.8 | 11.63 | <0.64 | 0.37 | 3.64 | | | 7.10 | | 35.87 | | 8.44 | | 28.13 | 1987.65 | | 186.02 | | | 38.03 |
| REC16 | 162.00 | <4.8 | <2.56 | 3.62 | 16.65 | <0.64 | 4.32 | 2.89 | | | <2.56 | | 28.79 | | 5.30 | | 12.29 | 6061.37 | | 519.72 | | | 3.67 |
| REC17 | 253.15 | <4.8 | <2.56 | 2.98 | 12.29 | <0.64 | 1.37 | 2.46 | | | <2.56 | | 25.42 | | 8.04 | | 3.14 | 4700.62 | | 186.02 | | | 22.82 |
| REC18 | 285.58 | 1434.98 | <2.56 | 3.99 | 13.17 | <0.64 | <0.64 | 2.54 | | | 163.28 | | <6.4 | | 14.43 | | 111.80 | 5448.59 | | 38.72 | | | 5.59 |
| REC19 | 69.90 | <4.8 | <2.56 | <4.8 | 2.39 | <0.64 | <0.64 | 5.25 | | | 1.88 | | 1.29 | | 7.30 | | 12.29 | 8080.52 | | 231.12 | | | 12.98 |
| REC21 | 19.84 | <4.8 | <2.56 | <4.8 | 4.25 | <0.64 | <0.64 | 1.53 | | | 0.95 | | <6.4 | | 9.64 | | 18.70 | 1745.11 | | 267.19 | | | 34.86 |
| REC22 | 93.69 | <4.8 | <2.56 | 0.93 | 2.88 | 0.30 | <0.64 | 17.68 | | | 17.10 | | <6.4 | | 14.90 | | <3.2 | 1624.17 | | 137.92 | | | 8.92 |
| REC23 | 68.60 | <4.8 | <2.56 | 3.98 | 25.26 | 0.76 | 1.61 | 4.99 | | | 2.47 | | 43.11 | | 12.30 | | 46.71 | 4324.68 | | 125.9 | | | 23.16 |
| REC24 | 157.55 | <4.8 | <2.56 | 1.26 | 3.34 | <0.64 | 0.30 | 5.38 | | | <2.56 | | 1.43 | | 4.22 | | <3.2 | 5568.49 | | 164.98 | | | 0.38 |
| REC25 | 79.18 | 36.76 | 0.92 | 13.03 | 46.33 | 3.27 | 2.81 | 13.48 | | | 19.72 | | 96.22 | | 21.14 | | 75.33 | 91.82 | | 255.17 | | | 34.63 |
| REC26 | 80.04 | <4.8 | <2.56 | 1.08 | 6.52 | <0.64 | 0.20 | 14.76 | | | 2.47 | | 6.95 | | 8.17 | | 28.98 | 4303.57 | | 288.24 | | | 32.76 |
| REC27 | 63.18 | <4.8 | <2.56 | <4.8 | 5.62 | <0.64 | <0.64 | 3.16 | | | <2.56 | | 14.57 | | 6.64 | | 7.17 | 4317.81 | | 8.65 | | | 19.68 |
| REC29 | 38.57 | 42.01 | <2.56 | 2.88 | 16.00 | <0.64 | 4.31 | 10.18 | | | 32.07 | | 25.42 | | 18.41 | | 28.13 | 1923.48 | | 219.09 | | | 40.75 |
| REC33 | 38.30 | <4.8 | <2.56 | 2.21 | 8.30 | <0.64 | <0.64 | 2.54 | | | 10.65 | | 5.45 | | 10.17 | | 12.70 | 1644.19 | | 246.15 | | | 10.64 |
| REC34 | 66.92 | 48066.4 | <2.56 | <4.8 | 9.65 | <0.64 | <0.64 | 5.42 | | | 1449.81 | | <6.4 | | 30.36 | | 171.99 | 7890.31 | | 4403.8 | | | 13.86 |
| REC35 | 50.56 | <4.8 | <2.56 | 1.45 | 17.74 | 0.34 | 1.92 | 13.56 | | | 0.95 | | 35.84 | | 9.51 | | 39.98 | 344.38 | | 600.89 | | | 3.39 |
| REC36 | 69.21 | <4.8 | <2.56 | <4.8 | 1.93 | <0.64 | <0.64 | 2.46 | | | 1.16 | | <6.4 | | 12.50 | | <3.2 | 348.80 | | 198.05 | | | 22.5 |
| REC37 | 81.34 | <4.8 | <2.56 | <4.8 | 8.31 | <0.64 | 0.62 | 5.94 | | | 3.82 | | 7.04 | | 7.51 | | 8.68 | 570.48 | | 186.02 | | | 11.05 |
| REC38 | 101.75 | <4.8 | <2.56 | 2.59 | 6.52 | <0.64 | 2.33 | 3.81 | | | 8.86 | | 26.87 | | 8.57 | | 13.15 | 5762.84 | | 540.76 | | | 4.55 |
| REC39 | 94.32 | <4.8 | <2.56 | 0.91 | 7.64 | <0.64 | 1.22 | 5.94 | | | <2.56 | | 18.30 | | 7.51 | | 12.70 | 1949.31 | | 95.83 | | | 14.85 |
| REC40 | 75.92 | <4.8 | <2.56 | <4.8 | 1.46 | <0.64 | 0.69 | 1.75 | | | <2.56 | | <6.4 | | 4.02 | | 11.32 | 3630.69 | | 201.05 | | | 9.97 |
| **Mean** | 98.63 | 1955.86 | 2.60 | 4.15 | 12.13 | 1.38 | 1.10 | 6.75 | | | 53.31 | | 20.23 | | 12.18 | | 29.16 | 2979.46 | | 372.67 | | | 17.37 |
| **SD** | 62.30 | 13585.45 | 0.51 | 4.65 | 13.04 | 3.74 | 1.05 | 6.96 | | | 244.59 | | 24.51 | | 8.36 | | 35.70 | 2475.54 | | 748.38 | | | 12.50 |
| **% Elevated** | 5.71 | 14.29 | 5.71 | 8.57 | 11.43 | 14.29 | 0.00 | 5.71 | | | 5.71 | | 5.71 | | 2.86 | | 11.43 | 11.43 | | 14.29 | | | 0.00 |
| Control Mean + 2xSD | 248.53 | 137.32 | 2.56 | 11.05 | 28.09 | 0.64 | 9.29 | 16.93 | | | 56.39 | | 44.99 | | 32.43 | | 73.36 | 6380.78 | | 438.08 | | | 106.54 |

^δ^ Concentration values in gray highlight are considered elevated above the normal control mean + 2xSD
