# Supplementary material for: Elevated cytokines and chemokines in peripheral blood of patients with SARS-CoV-2 pneumonia treated with high-titer convalescent plasma
Source: PLoS Pathog. 2021 Oct 29;17(10):e1010025. doi: 10.1371/journal.ppat.1010025 (PMC8580259; doi:10.1371/journal.ppat.1010025)
Supplement: S4 Table — (DOCX) [file ppat.1010025.s005.docx]

| **S4 Table. Recipient Plasma Luminex Analyses**  **Day 10 Concentration (pg/ml) of Analytes with Elevation 0-19% of Recipients ^δ^** | | | | | | | | |  |  |  | |  |  | |  |  |  |  |
| --- | --- | --- | --- | --- | --- | --- | --- | --- | --- | --- | --- | --- | --- | --- | --- | --- | --- | --- | --- |
| **Recipient** | **Eotaxin (CCL11)** | **G-CSF** | **GM-CSF** | **IL-1α** | **IL-1β** | **IL-2** | **IL-4** | **IL-5** | **IL-10** | **IL-13** | | **IL-15** | | | **MIP-1a (CCL3)** | | **MBL ng/ml** | **PCT ng/ml** | **SP-D** |
| REC01 | 72.10 | <4.8 | <2.56 | 0.19 | 24.55 | <0.64 | 0.14 | 2.92 | <2.56 | 17.25 | | 5.98 | | | 14.35 | | 2595.66 | 135.95 | 6.58 |
| REC02 | 366.79 | <4.8 | <2.56 | 0.64 | 5.98 | <0.64 | 0.36 | 2.81 | <2.56 | 5.45 | | 8.40 | | | <3.2 | | 155.43 | 85.12 | 9.22 |
| REC03 * | - | - | - | - | - | - | - | - | - | - | | - | | | - | | - | - | - |
| REC04 | 151.69 | <4.8 | <2.56 | <4.8 | 8.26 | <0.64 | 1.89 | 10.16 | <2.56 | 33.10 | | 4.67 | | | 20.79 | | 626.90 | 213.09 | 11.71 |
| REC05 | 211.08 | <4.8 | <2.56 | <4.8 | 10.51 | <0.64 | 1.12 | 3.61 | 1.01 | 8.80 | | 6.66 | | | 21.21 | | 2669.21 | 248.14 | 30.19 |
| REC06 | 173.55 | <4.8 | <2.56 | 3.75 | 9.02 | 1.56 | 1.56 | 2.77 | <2.56 | 6.57 | | 6.42 | | | 16.56 | | 1930.16 | 83.36 | 2.74 |
| REC07 | 140.83 | <4.8 | <2.56 | <4.8 | 7.84 | <0.64 | <0.64 | 0.86 | 4.64 | 6.4 | | 12.40 | | | 10.68 | | 1250.73 | 153.48 | 5.91 |
| REC08 | 144.55 | 19.67 | <2.56 | 1.30 | 4.47 | 8.62 | 0.08 | 28.16 | 141.33 | 6.4 | | 66.79 | | | 58.87 | | 617.72 | 2573.5 | 7.89 |
| REC09 | 137.41 | <4.8 | <2.56 | <4.8 | 1.84 | <0.64 | <0.64 | 5.71 | <2.56 | 6.4 | | 10.55 | | | <3.2 | | 5807.12 | 196.43 | 16.93 |
| REC10 | 243.54 | 982.62 | <2.56 | 1.31 | 13.15 | <0.64 | 1.94 | 1.55 | 45.62 | 6.4 | | 34.90 | | | 47.28 | | 551.51 | 202.57 | 48.96 |
| REC11 | 156.13 | <4.8 | <2.56 | <4.8 | 5.13 | <0.64 | 0.25 | 6.79 | <2.56 | <6.4 | | 21.94 | | | 6.85 | | 1676.92 | 159.62 | 48.54 |
| REC12 * | - | - | - | - | - | - | - | - | - | - | | - | | | - | | - | - | - |
| REC13 | 163.82 | 59.43 | <2.56 | <4.8 | 5.74 | <0.64 | 1.17 | 2.39 | 2.74 | 22.56 | | 8.14 | | | 20.62 | | 2210.13 | 128.07 | 26.17 |
| REC14 | 133.52 | <4.8 | <2.56 | <4.8 | 4.05 | <0.64 | <0.64 | 1.55 | <2.56 | <6.4 | | 4.55 | | | <3.2 | | 812.44 | 112.29 | 19.71 |
| REC15 | 217.54 | 5.75 | <2.56 | 2.50 | 6.59 | <0.64 | <0.64 | 4.15 | <2.56 | 19.03 | | 3.35 | | | 21.21 | | 1647.43 | 152.61 | 36.75 |
| REC16 | 91.34 | <4.8 | <2.56 | <4.8 | 4.91 | <0.64 | 2.11 | 2.01 | <2.56 | 2.05 | | 2.40 | | | <3.2 | | 5969.74 | 181.53 | 4.80 |
| REC17 | 95.18 | <4.8 | <2.56 | <4.8 | <1.6 | <0.64 | <0.64 | 4.76 | <2.56 | <6.4 | | 5.17 | | | <3.2 | | 9782.93 | 86.87 | 15.65 |
| REC18 | 34.87 | 15240.9 | <2.56 | 4.45 | 8.67 | <0.64 | <0.64 | 1.36 | 10.11 | <6.4 | | 43.24 | | | 18.56 | | 4342.28 | 1728.5 | 36.61 |
| REC19 | 477.64 | 4.40 | <2.56 | 1.78 | 3.17 | <0.64 | <0.64 | 51.10 | <2.56 | <6.4 | | 6.35 | | | 36.47 | | 4633.79 | 164.00 | 30.00 |
| REC21 | 24.75 | <4.8 | <2.56 | <4.8 | 2.75 | <0.64 | <0.64 | 1.89 | 39.52 | <6.4 | | 27.80 | | | 7.73 | | 1762.13 | 417.31 | 26.44 |
| REC22 | 339.49 | 253.04 | <2.56 | 2.66 | 7.44 | 0.27 | <0.64 | 44.36 | 7.42 | <6.4 | | 10.12 | | | 48.02 | | 1078.19 | 68.46 | 16.60 |
| REC23 | 200.23 | <4.8 | <2.56 | 1.88 | 16.13 | 0.13 | 0.63 | 16.71 | <2.56 | 6.55 | | 7.22 | | | 24.80 | | 3877.32 | 170.14 | 30.41 |
| REC24 | 105.96 | <4.8 | <2.56 | <4.8 | <1.6 | <0.64 | <0.64 | 1.32 | <2.56 | <6.4 | | 1.96 | | | <3.2 | | 6140.05 | 201.69 | 0.21 |
| REC25 | 88.24 | 6.35 | <2.56 | <4.8 | 17.49 | 0.19 | 0.85 | 4.80 | 1.55 | 24.93 | | 8.95 | | | 31.99 | | 154.54 | 174.52 | 35.14 |
| REC26 | 57.78 | 33.51 | <2.56 | <4.8 | 12.34 | <0.64 | 1.78 | 2.16 | <2.56 | 20.33 | | 9.19 | | | 34.32 | | 141.11 | 75.48 | 0.06 |
| REC27 | 232.75 | <4.8 | <2.56 | <4.8 | 2.28 | <0.64 | <0.64 | 3.31 | <2.56 | 3.27 | | 2.84 | | | <3.2 | | 3129.74 | 97.39 | 19.81 |
| REC29 | 204.45 | 9.66 | <2.56 | 0.19 | 12.35 | <0.64 | 2.99 | 7.02 | 17.38 | 11.24 | | 10.31 | | | 10.68 | | 1540.93 | 123.68 | 47.65 |
| REC33 | 57.67 | <4.8 | <2.56 | <4.8 | <1.6 | <0.64 | <0.64 | 1.13 | <2.56 | <6.4 | | 3.35 | | | <3.2 | | 1581.89 | 128.07 | 5.41 |
| REC34 * | - | - | - | - | - | - | - | - | - | - | | - | | | - | | - | - | - |
| REC35 | 136.29 | <4.8 | <2.56 | <4.8 | 4.05 | <0.64 | <0.64 | 2.31 | <2.56 | 17.76 | | 5.61 | | | 16.54 | | 118.57 | 127.19 | 4.49 |
| REC36 | 173.13 | <4.8 | <2.56 | <4.8 | 2.71 | <0.64 | <0.64 | 2.08 | 4.27 | <6.4 | | 9.38 | | | <3.2 | | 530.49 | 95.64 | 7.13 |
| REC37 | 135.59 | <4.8 | <2.56 | <4.8 | 2.30 | 0.<0.64 | <0.64 | 7.37 | <2.56 | <6.4 | | 3.10 | | | <3.2 | | 869.54 | 99.14 | 16.11 |
| REC38 | 229.32 | 28.72 | <2.56 | <4.8 | <1.6 | <0.64 | <0.64 | 2.24 | <2.56 | <6.4 | | 6.48 | | | <3.2 | | 5021.19 | 167.51 | 8.52 |
| REC39 | 235.82 | <4.8 | <2.56 | 0.55 | 15.15 | 0.20 | 0.90 | 7.71 | 1.83 | 31.12 | | 10.31 | | | 34.52 | | 2999.90 | 210.46 | 18.39 |
| REC40 | 293.35 | 15.38 | <2.56 | <4.8 | <1.6 | <0.64 | <0.64 | 1.70 | <2.56 | <6.4 | | 5.55 | | | 13.09 | | 3926.36 | 135.08 | 20.31 |
| **Mean** | 172.70 | 520.97 | 2.56 | 3.66 | 7.09 | 0.86 | 0.89 | 7.46 | 10.26 | 10.59 | | 11.69 | | | 17.20 | | 2504.75 | 278.03 | 19.22 |
| **SD** | 99.00 | 2691.87 | 0 | 1.70 | 5.64 | 1.43 | 0.63 | 11.88 | 25.96 | 8.08 | | 13.72 | | | 15.40 | | 2260.14 | 507.26 | 14.38 |
| **% Elevated** | 12.50 | 9.38 | 0.00 | 0.00 | 0.00 | 6.25 | 0.00 | 9.38 | 3.13 | 0.00 | | 9.38 | | | 0.00 | | 3.13 | 9.38 | 0.00 |
| **Control Mean + 2xSD** | 248.53 | 137.32 | 2.56 | 11.05 | 28.09 | 0.64 | 9.29 | 16.93 | 56.39 | 44.99 | | 32.43 | | | 73.36 | | 6380.78 | 438.08 | 106.54 |

^^ Concentration values in gray highlight are considered elevated above the normal control mean + 2xSD

* Recipient was deceased by Day 10
