# Supplementary material for: Elevated cytokines and chemokines in peripheral blood of patients with SARS-CoV-2 pneumonia treated with high-titer convalescent plasma
Source: PLoS Pathog. 2021 Oct 29;17(10):e1010025. doi: 10.1371/journal.ppat.1010025 (PMC8580259; doi:10.1371/journal.ppat.1010025)
Supplement: S5 Table — (DOCX) [file ppat.1010025.s006.docx]

| **Sample** | **EGF** | **Eotaxin** | **G-CSF** | **GM-CSF** | **IFNα2** | **IFNγ** | **IL-1α** | **IL-1β** | **IL-1RA** | **IL-2** | **IL-3** | **IL-4** | **IL-5** | **IL-6** | **IL-7** | **IL-8** | **IL-10** | **IL-12p40** |
| --- | --- | --- | --- | --- | --- | --- | --- | --- | --- | --- | --- | --- | --- | --- | --- | --- | --- | --- |
| **Norm 1** | 3.20 | 94.81 | 4.80 | 2.56 | 38.66 | 18.57 | 4.80 | 8.95 | 2.00 | 0.64 | 1.28 | 0.64 | 3.30 | 0.77 | 0.64 | 1.55 | 2.56 | 23.03 |
| **Norm 2** | 3.20 | 190.03 | 4.80 | 2.56 | 28.89 | 8.66 | 4.80 | 3.20 | 4.73 | 0.64 | 1.28 | 0.64 | 1.75 | 0.64 | 0.64 | 1.67 | 2.56 | 8.18 |
| **Norm 3** | 12.98 | 95.16 | 4.80 | 2.56 | 40.26 | 5.51 | 4.80 | 5.56 | 3.16 | 0.64 | 1.28 | 0.64 | 1.54 | 0.64 | 0.64 | 1.81 | 2.56 | 19.27 |
| **Norm 4** | 30.96 | 28.92 | 165.01 | 2.56 | 35.42 | 22.43 | 11.87 | 31.17 | 13.15 | 0.64 | 1.28 | 11.56 | 17.26 | 0.64 | 0.64 | 0.78 | 68.27 | 36.45 |
| **Norm 5** | 26.09 | 30.88 | 4.80 | 2.56 | 35.47 | 4.77 | 4.80 | 11.74 | 6.12 | 0.64 | 1.28 | 0.64 | 8.44 | 0.97 | 0.64 | 0.73 | 2.56 | 33.22 |
| **Norm 6** | 22.82 | 40.67 | 58.34 | 2.56 | 30.33 | 25.31 | 10.92 | 25.43 | 15.10 | 0.64 | 1.28 | 4.84 | 16.77 | 0.64 | 0.64 | 0.67 | 37.14 | 42.08 |
| **Norm 7** | 21.75 | 75.23 | 108.73 | 2.56 | 11.95 | 1.28 | 4.80 | 3.11 | 1.77 | 0.64 | 1.28 | 6.16 | 2.94 | 0.64 | 0.64 | 0.64 | 33.91 | 17.18 |
| **Norm 8** | 3.20 | 38.04 | 4.80 | 2.56 | 8.00 | 1.28 | 4.80 | 2.63 | 1.60 | 0.64 | 1.28 | 0.64 | 2.37 | 0.64 | 0.64 | 0.64 | 2.56 | 29.49 |
| **Norm 9** | 3.20 | 89.92 | 4.80 | 2.56 | 30.25 | 1.28 | 4.80 | 3.11 | 2.53 | 0.64 | 1.28 | 0.64 | 3.64 | 0.64 | 0.64 | 0.64 | 2.56 | 26.59 |
| **Norm 11** | 12.58 | 171.22 | 4.80 | 2.56 | 8.00 | 1.28 | 4.80 | 1.60 | 12.67 | 0.64 | 1.28 | 0.64 | 0.70 | 0.64 | 0.64 | outlier | 2.56 | 6.40 |
| **Norm 12** | 3.20 | 270.18 | 4.80 | 2.56 | 8.00 | 1.28 | 4.80 | 3.63 | 50.16 | 0.64 | 1.28 | 0.64 | 0.93 | 0.64 | 0.64 | 0.64 | 2.56 | 6.40 |
| **Norm 13** | 3.20 | 58.82 | 4.80 | 2.56 | 8.64 | 1.28 | 4.80 | 1.86 | 1.60 | 0.64 | 1.28 | 1.20 | 2.31 | 0.64 | 0.64 | 2.40 | 2.56 | 10.50 |
| **Mean** | 12.20 | 98.66 | 31.27 | 2.56 | 23.66 | 7.74 | 5.90 | 8.50 | 9.55 | 0.64 | 1.28 | 2.41 | 5.16 | 0.68 | 0.64 | 1.11 | 13.53 | 21.57 |
| **SD** | 10.60 | 74.94 | 53.02 | 0.00 | 13.46 | 9.08 | 2.58 | 9.80 | 13.72 | 0.00 | 0.00 | 3.44 | 5.89 | 0.10 | 0.00 | 0.63 | 21.43 | 12.26 |
| **Control Mean + 2xSD** | 33.40 | 248.53 | 137.32 | 2.56 | 50.57 | 25.90 | 11.05 | 28.09 | 37.00 | 0.64 | 1.28 | 9.29 | 16.93 | 0.87 | 0.64 | 2.37 | 56.39 | 46.10 |

**S5 Table. Plasma Concentration of Analytes in Normal Controls***

| **Sample** | **IL-12p70** | **IL-13** | **IL-15** | **IL-17A** | **MCP-1** | **MIP-1α** | **MIP-1β** | **RANTES** | **TNFα** | **TNFβ** | **VEGF** | **IP-10** | **MBL (ng/ml)** | **CRP (ug/ml)** | **PCT** | **NGAL (ng/ml)** | **SP-D** |
| --- | --- | --- | --- | --- | --- | --- | --- | --- | --- | --- | --- | --- | --- | --- | --- | --- | --- |
| **Norm 1** | 3.20 | 19.68 | 6.56 | 6.20 | 302.76 | 4.04 | 18.22 | 2820.32 | 11.19 | 1.60 | 2.56 | 11.30 | 4328.30 | 0.80 | - | - | - |
| **Norm 2** | 3.20 | 6.40 | 3.43 | 1.28 | 320.84 | 32.99 | 17.22 | 3499.92 | 12.52 | 1.60 | 13.07 | 17.02 | 3650.10 | 0.07 | - | - | - |
| **Norm 3** | 3.20 | 6.40 | 3.65 | 4.17 | 277.20 | 3.20 | 20.08 | 4664.29 | 9.55 | 1.60 | 10.81 | 68.45 | 1283.50 | 4.81 | - | - | - |
| **Norm 4** | 3.20 | 37.02 | 32.58 | 13.64 | 115.23 | 72.18 | 22.78 | 2766.06 | 13.56 | 11.49 | 49.06 | 149.03 | outlier | 1.00 | 12.25 | 48.30 | 68.89 |
| **Norm 5** | 3.93 | 18.56 | 14.10 | 7.40 | 143.26 | 34.67 | 22.29 | 2766.73 | 12.04 | 1.60 | 2.56 | 98.18 | 5130.36 | outlier | 43.00 | 66.60 | 74.22 |
| **Norm 6** | 3.20 | 52.18 | 33.65 | 15.52 | 130.64 | 70.68 | 26.09 | 4447.13 | 42.47 | 3.41 | 32.38 | 84.27 | 5616.73 | 0.01 | 64.32 | 67.30 | 45.37 |
| **Norm 7** | 3.2 | 6.40 | 4.15 | 1.28 | 198.91 | 3.20 | 9.90 | 2427.33 | 6.40 | 1.60 | 36.42 | 91.73 | 3731.41 | 6.97 | 435.54 | 126.20 | 43.36 |
| **Norm 8** | 3.2 | 6.40 | 3.20 | 1.58 | 159.57 | 3.20 | 23.36 | 3004.89 | 6.40 | 1.60 | 2.56 | 47.82 | 1675.06 | 2.93 | 228.11 | 73.66 | 1.33 |
| **Norm 9** | 3.20 | 6.61 | 3.20 | 3.59 | 169.00 | 3.20 | 16.89 | 3539.96 | 10.57 | 1.60 | 20.28 | 65.67 | 765.91 | 4.01 | 77.80 | 102.89 | 7.77 |
| **Norm 10** | - | - | - | - | - | - | - | - | - | - | - | - | - | - | 141.93 | 71.23 | - |
| **Norm 11** | 3.20 | 6.40 | 3.20 | 1.50 | 159.58 | 3.20 | 10.50 | 1949.63 | 6.60 | 1.60 | 36.81 | 64.63 | 2019.63 | 3.50 | - | 352.11 | - |
| **Norm 12** | 3.20 | 6.40 | 3.20 | 1.28 | 239.07 | 3.20 | 2.29 | 1949.31 | 6.40 | 1.60 | 19.45 | 56.60 | 3147.51 | 7.00 | - | 220.90 | - |
| **Norm 13** | 3.20 | 6.40 | 3.20 | 1.28 | 137.34 | 3.20 | 4.55 | 1890.71 | 6.40 | 1.60 | 40.26 | 92.82 | 677.79 | 0.76 | - | 94.08 | - |
| **Norm 14** | - | - | - | - | - | - | - | - | - | - | - | - | - | - | - | 90.13 | - |
| **Norm 15** | - | - | - | - | - | - | - | - | - | - | - | - | - | - | 330.73 | - | - |
| **Norm 16** | - | - | - | - | - | - | - | - | - | - | - | - | - | - | 258.03 | - | - |
| **Norm 17** | - | - | - | - | - | - | - | - | - | - | - | - | - | - | 393.33 | - | - |
| **Norm 18** | - | - | - | - | - | - | - | - | - | - | - | - | - | - | - | 366.45 | 5.32 |
| **Norm 19** | - | - | - | - | - | - | - | - | - | - | - | - | - | - | - | outlier | 6.77 |
| **Norm 20** | - | - | - | - | - | - | - | - | - | - | - | - | - | - | - | 325.39 | 11.85 |
| **Norm 21** | - | - | - | - | - | - | - | - | - | - | - | - | - | - | - | 68.88 | 12.57 |
| **Mean** | 3.26 | 14.90 | 9.51 | 4.89 | 196.12 | 19.75 | 16.18 | 2977.19 | 12.01 | 2.57 | 22.18 | 70.63 | 2911.48 | 2.90 | 198.50 | 148.14 | 27.74 |
| **SD** | 0.21 | 15.04 | 11.46 | 4.99 | 71.25 | 26.81 | 7.70 | 919.80 | 9.97 | 2.85 | 16.37 | 37.18 | 1734.65 | 2.60 | 153.01 | 116.26 | 27.75 |
| **Control Mean + 2xSD** | 3.68 | 44.99 | 32.43 | 14.88 | 338.62 | 73.36 | 31.57 | 4816.80 | 31.96 | 8.28 | 54.93 | 144.98 | 6380.78 | 8.10 | 504.51 | 380.66 | 83.24 |

**S5 Table. Plasma Concentration of Analytes in Normal Controls (cont.)**

| **Sample** | **IgM (ug/ml)** | **IgG1 (ug/ml)** | **IgG2 (ug/ml)** | **IgG3 (ug/ml)** | **IgG4 (ug/ml)** | **IgA (ug/ml)** |
| --- | --- | --- | --- | --- | --- | --- |
| **Norm 18** | 1362.53 | 2599.26 | 1061.51 | 421.64 | 96.04 | 694.19 |
| **Norm 19** | 636.66 | 1703.80 | 737.03 | 149.96 | 8.03 | 352.81 |
| **Norm 20** | 941.51 | 1299.57 | 1502.17 | 320.17 | 229.54 | 683.53 |
| **Norm 21** | 1176.33 | 3500.52 | 2220.19 | 475.87 | 645.98 | 767.28 |
| **Norm 4** | 670.97 | 4233.65 | 2312.06 | 877.74 | 363.33 | 1359.24 |
| **Norm 5** | 728.09 | 4754.90 | 2587.41 | outlier | 110.17 | 1988.49 |
| **Norm 6** | 361.14 | 2898.24 | 944.18 | 508.97 | - | 1329.11 |
| **Norm 7** | 954.25 | 1917.82 | 1208.89 | 335.03 | 272.04 | 332.10 |
| **Norm 8** | 1010.71 | 3026.69 | 1387.85 | 363.11 | 22.59 | 841.27 |
| **Norm 9** | 452.16 | 3269.18 | 480.93 | 431.81 | 322.63 | 473.78 |
| **Norm 10** | 272.09 | 1886.73 | 1577.86 | 45.33 | 165.33 | 1454.48 |
| **Norm 11** | 482.51 | 1633.13 | - | 764.31 | 107.96 | 260.25 |
| **Norm 12** | 309.17 | 1841.95 | 1076.13 | 883.93 | 727.06 | 691.15 |
| **Norm 13** | 312.32 | 1090.92 | 1747.44 | 117.92 | 255.68 | 172.62 |
| **Mean** | 690.75 | 2546.88 | 1449.51 | 438.14 | 255.88 | 814.31 |
| **SD** | 350.77 | 1111.41 | 632.03 | 270.06 | 220.58 | 532.07 |
| **Control Mean + 2xSD** | 1392.28 | 4769.69 | 2713.58 | 978.25 | 697.03 | 1878.46 |

For purpose of calculating the mean, the Limit of Detection (LoD) values were used when the actual value was <LoD and extrapolated values were adjusted to LoD.

Any value with a z score +3/-3 was deemed an outlier and eliminated (highlighted in gray).

*Plasma concentration is in pg/ml unless otherwise noted.
