# Supplementary material for: Elevated cytokines and chemokines in peripheral blood of patients with SARS-CoV-2 pneumonia treated with high-titer convalescent plasma
Source: PLoS Pathog. 2021 Oct 29;17(10):e1010025. doi: 10.1371/journal.ppat.1010025 (PMC8580259; doi:10.1371/journal.ppat.1010025)
Supplement: S6 Table — (DOCX) [file ppat.1010025.s007.docx]

**S6 Table. Statistical Analyses** **of Comparison Between Time Points of Mean Concentrations of Cytokines/Chemokines**

|  | **TRACK 2** | | | **TRACK 3** | | | **TRACK 2 vs TRACK 3** | | |
| --- | --- | --- | --- | --- | --- | --- | --- | --- | --- |
| **Analyte** | **Day 0 vs Day 3** | **Day 0 vs Day 10** | **Day 3 vs Day 10** | **Day 0 vs Day 3** | **Day 0 vs Day 10** | **Day 3 vs Day 10** | **Day 0** | **Day 3** | **Day 10** |
| EGF | 0.2539 | **0.0185** | 0.2316 | 0.6823 | 0.6558 | 0.9945 | 0.7415 | 0.6391 | 0.0691 |
| Exotaxin | 0.1853 | **<0.0001** | **0.0036** | 0.6483 | **0.0413** | 0.0687 | 0.3798 | 0.3265 | 0.7736 |
| G-CSF | 0.8426 | 0.3516 | 0.2918 | 0.3243 | 0.3438 | 0.5527 | 0.3064 | 0.4305 | 0.3610 |
| GM-CSF | 0.3546 | 0.3282 | 0.2167 | - | - | - | 0.3282 | 0.2167 | - |
| IFNα2 | 0.1668 | **0.0006** | **0.0377** | 0.6014 | 0.6865 | 0.3740 | **0.0006** | 0.0957 | 0.5413 |
| IFNγ | **0.0009** | **0.0005** | 0.3863 | 0.2029 | 0.5359 | 0.1125 | **0.0094** | 0.2100 | 0.1334 |
| IL-1α | 0.8652 | **0.0164** | 0.1176 | 0.3320 | 0.3320 | - | 0.9028 | 0.1176 | - |
| IL-1β | 0.7628 | **0.0282** | **0.0446** | 0.9324 | 0.7333 | 0.7116 | 0.1948 | 0.1611 | 0.6897 |
| IL-1RA | 0.5252 | 0.2061 | 0.2171 | 0.0687 | **0.0136** | 0.7410 | **0.0382** | 0.1406 | 0.3835 |
| IL-2 | 0.8448 | 0.4191 | 0.4394 | 0.8356 | 0.3388 | 0.1911 | 0.2660 | 0.2312 | 0.2779 |
| IL-3 | **0.0201** | **0.0010** | 0.2097 | 0.3388 | - | 0.3388 | **0.0007** | 0.1836 | 0.3287 |
| IL-4 | 0.2731 | 0.7739 | 0.1874 | 0.8732 | 0.8957 | 0.9663 | 0.5002 | 0.8133 | 0.4071 |
| IL-5 | 0.3391 | 0.5056 | 0.8700 | 0.1601 | 0.2385 | 0.7789 | 0.2275 | 0.5291 | 0.5545 |
| IL-6 | 0.1928 | 0.1159 | 0.5939 | 0.1652 | 0.1152 | 0.4909 | 0.1369 | 0.1831 | 0.1852 |
| IL-7 | 0.7400 | 0.4929 | 0.4171 | 0.1104 | 0.1328 | 0.7794 | 0.4773 | 0.1038 | 0.1788 |
| IL-8 | 0.4590 | 0.3899 | 0.7340 | 0.2812 | 0.3742 | 0.4371 | 0.2896 | 0.2789 | 0.3926 |
| IL-10 | **0.0168** | 0.7016 | 0.5523 | 0.2981 | 0.5761 | 0.2880 | 0.5994 | 0.2827 | 0.7431 |
| IL-12p40 | 0.1186 | **0.0205** | 0.1996 | 0.9910 | 0.7287 | 0.6924 | **0.0035** | **0.0087** | 0.1488 |
| IL-12p70 | 0.6278 | **0.0059** | **0.0856** | 0.5690 | 0.2235 | 0.2106 | **0.0052** | 0.1093 | 0.0905 |
| IL-13 | 0.7150 | **0.0028** | **0.0346** | 0.4552 | 0.3051 | 0.6361 | 0.3468 | 0.1030 | 0.9612 |
| IL-15 | 0.5696 | 0.4298 | 0.7076 | 0.5703 | 0.4894 | 0.7346 | 0.0577 | 0.7408 | 0.6983 |
| IL-17A | 0.4956 | **0.0364** | 0.1420 | 0.8996 | 0.6830 | 0.7699 | **0.0268** | 0.1245 | 0.8796 |
| MCP-1 | 0.3274 | 0.5423 | 0.2765 | 0.4821 | 0.8899 | 0.5735 | 0.7935 | 0.5231 | 0.4060 |
| MIP-1α | 0.4559 | 0.0604 | 0.2816 | 0.1210 | 0.5546 | 0.1736 | **0.0146** | 0.3760 | 0.8090 |
| MIP-1β | 0.9684 | 0.3007 | 0.2909 | 0.1584 | 0.3792 | 0.2546 | 0.5857 | 0.1844 | 0.8192 |
| RANTES | 0.2419 | **0.0425** | **0.0098** | **0.0283** | **0.0073** | 0.0645 | 0.6239 | 0.1657 | 0.4307 |
| TNFα | 0.6500 | 0.5312 | 0.6687 | 0.1518 | **0.0537** | 0.4143 | **0.0015** | 0.2772 | 0.7672 |
| TNFβ | 0.7332 | 0.1142 | 0.2693 | 0.1962 | 0.1013 | 0.9560 | 0.1401 | 0.6064 | 0.0915 |
| VEGF | 0.4515 | **0.0189** | 0.0785 | 0.5033 | 0.2139 | 0.0740 | 0.2500 | 0.9716 | 0.4842 |
| IP-10 | 0.4011 | 0.1818 | 0.5804 | 0.6492 | 0.3208 | 0.2980 | 0.6503 | 0.7227 | 0.3001 |
| MBL | 0.9673 | 0.8437 | 0.7967 | 0.4455 | 0.4452 | 0.1059 | 0.9969 | 0.3234 | 0.2189 |
| CRP | 0.5496 | **0.0216** | **0.0210** | 0.3588 | 0.3532 | 0.1187 | 0.8134 | 0.5724 | 0.7115 |
| NGAL | 0.2805 | **<0.0001** | **<0.0001** | 0.7260 | **0.0213** | **0.0169** | 0.2332 | 0.3309 | 0.4616 |
| PCT | **0.0003** | 0.1210 | 0.7667 | 0.1594 | 0.2362 | 0.4044 | 0.4311 | 0.2570 | 0.7653 |
| SP-D | 0.0953 | 0.3036 | 0.5012 | 0.0711 | 0.1041 | 0.9172 | 0.1110 | 0.2085 | 0.4982 |
