# Supplementary material for: Elevated cytokines and chemokines in peripheral blood of patients with SARS-CoV-2 pneumonia treated with high-titer convalescent plasma
Source: PLoS Pathog. 2021 Oct 29;17(10):e1010025. doi: 10.1371/journal.ppat.1010025 (PMC8580259; doi:10.1371/journal.ppat.1010025)
Supplement: S7 Table — (DOCX) [file ppat.1010025.s008.docx]

**S7 Table. Pre-Infusion Individual Recipient Cytokine Levels of Interest**

| **Recipient** | **EGF** | **IFNα2** | **IFNγ** | **IL-1RA** | **IL-3** | **IL-6** | **IL-7** | | **IL-8 (CXCL8)** | **IL-12p40** | **IL-12p70** | **IL-17A** | **IP-10 (CXCL10)** | | **MCP-1 (CCL2)** | **MIP-1β (CCL4)** | **RANTES (CCL5)** | **TNFα** | **TNFβ** | **VEGF** | **CRP ug/ml** | | **NGAL ng/ml** |
| --- | --- | --- | --- | --- | --- | --- | --- | --- | --- | --- | --- | --- | --- | --- | --- | --- | --- | --- | --- | --- | --- | --- | --- |
| **TRACK 2** | | | | | | | | | | | | | | | | | | | | | | | |
| REC01 | 8.16 | 51.53 | 50.10 | 21.01 | 2.04 | 40.17 | | 2.14 | 2.49 | 34.84 | 4.49 | 11.39 | | 1100.55 | 292.39 | 30.88 | 4150.95 | 23.17 | 3.15 | <2.56 | 585.64 | | 586.3 |
| REC03 | 15.99 | 91.26 | 39.31 | 15.48 | 4.56 | 4.35 | | 2.15 | 8.93 | 58.84 | 10.43 | 17.09 | | 440.98 | 898.85 | 52.04 | 4043.38 | 44.23 | 11.49 | 41.14 | 26.13 | | 175.8 |
| REC05 | 6.94 | 70.83 | 44.74 | 10.18 | 5.34 | 4.91 | | 42.90 | 4.47 | 37.26 | 5.23 | 16.47 | | 728.59 | 684.89 | 47.80 | 3851.95 | 33.66 | 9.31 | 33.16 | 92.36 | | 237.1 |
| REC06 | 14.49 | 77.04 | 189.13 | 27.55 | 2.72 | 31.12 | | 15.36 | 12.07 | 68.35 | 7.13 | 75.73 | | 1430.77 | 1144.92 | 44.85 | 5386.93 | 34.05 | 12.13 | 267.85 | 559.44 | | 400.6 |
| REC07 | 3.74 | 27.48 | <1.28 | 5.41 | 0.88 | 9.76 | | <0.64 | 3.58 | 11.07 | 1.56 | 1.90 | | 1015.07 | 599.93 | 34.92 | 1321.56 | 15.86 | <1.6 | <2.56 | 83.11 | | 124.7 |
| REC08 | 7.81 | 57.56 | 35.56 | 32.01 | 2.09 | 8.48 | | 0.41 | 9.63 | 28.75 | 6.94 | 14.23 | | 859.19 | 495.22 | 66.24 | 3001.40 | 30.17 | 6.23 | 33.36 | 173.30 | | 164.7 |
| REC09 | 10.17 | 86.28 | 64.11 | 12.20 | 3.49 | 11.43 | | 13.05 | 5.02 | 51.67 | 10.80 | 16.88 | | 1399.83 | 432.42 | 39.06 | 3171.49 | 33.49 | 9.43 | 16.75 | 128.92 | | 119.2 |
| REC10 | 5.20 | 22.08 | 8.77 | 3.35 | <1.28 | 62.99 | | <0.64 | 33.70 | 321.69 | 0.41 | <1.28 | | 18957.73 | 862.47 | 176.16 | 488.09 | 89.44 | <1.6 | <2.56 | 312.43 | | 23.7 |
| REC11 | <3.2 | 1.39 | <1.28 | 0.77 | <1.28 | 63.07 | | <0.64 | 46.13 | 11.07 | <3.2 | <1.28 | | 2395.42 | 1358.71 | 13.35 | 147.11 | 16.62 | <1.6 | <2.56 | 480.18 | | 75.3 |
| REC13 | 14.38 | 118.75 | 193.56 | 20.64 | 5.92 | 15.21 | | 2.96 | 5.25 | 171.72 | 17.86 | 19.50 | | 2484.24 | 508.60 | 48.36 | 4164.39 | 73.54 | 10.33 | 22.86 | 385.08 | | 353.0 |
| REC15 | 13.48 | 74.96 | 44.22 | 7.20 | 2.91 | 8.35 | | 1.26 | 3.28 | 46.88 | 8.64 | 15.53 | | 1946.24 | 231.56 | 34.50 | 3497.73 | 28.22 | 5.72 | 9.81 | 519.71 | | 377.2 |
| REC16 | 15.59 | 34.20 | 17.53 | 6.63 | 1.70 | 0.58 | | 2.17 | 2.70 | 17.30 | 3.36 | 10.74 | | 55.74 | 186.67 | 30.54 | 4256.60 | 19.51 | 10.08 | 272.71 | 45.57 | | 334.8 |
| REC17 | 18.29 | 51.04 | 84.96 | 8.41 | 1.94 | 5.81 | | 2.55 | 7.22 | 48.49 | 5.25 | 11.72 | | 2318.89 | 270.94 | 30.32 | 3213.01 | 23.94 | 0.83 | 33.94 | 299.05 | | 377.8 |
| REC22 | 17.19 | 6.04 | <1.28 | 5.60 | <1.28 | 33.55 | | 12.88 | 48.94 | 5.99 | 0.50 | 1.05 | | 738.11 | 6568.98 | 24.67 | 17788.84 | 8.25 | 1.09 | 166.25 | 256.18 | | 492.7 |
| REC24 | 22.56 | 108.50 | 56.19 | 13.34 | 5.24 | 8.12 | | 2.85 | 3.87 | 94.66 | 15.22 | 21.91 | | 768.12 | 167.19 | 40.50 | 3682.81 | 45.22 | 16.60 | 13.15 | 127.27 | | 101.2 |
| REC25 | 61.85 | 124.06 | 66.64 | 15.98 | 6.63 | 2.59 | | 6.74 | 6.67 | 68.34 | 14.47 | 31.89 | | 327.93 | 164.02 | 44.31 | 2679.24 | 41.88 | 27.44 | 248.86 | 58.36 | | 317.1 |
| REC27 | 7.23 | <8 | 9.26 | 12.58 | <1.28 | 34.45 | | 1.68 | 11.78 | 21.42 | <3.2 | <1.28 | | 530.36 | 815.46 | 44.52 | 18549.42 | 2.65 | <1.6 | 0.82 | 228.21 | | 162.9 |
| REC33 | 9.65 | 8.02 | 158.09 | 18.25 | <1.28 | 20.47 | | 8.65 | 15.40 | 42.08 | 1.08 | 1.46 | | 40124.31 | 633.32 | 25.71 | 6673.61 | 18.93 | <1.6 | 2.11 | 1033.63 | | 197.1 |
| REC35 | 18.35 | 53.29 | 10.89 | 11.45 | 2.48 | 8.32 | | 1.92 | 8.41 | 28.36 | 1.94 | 9.42 | | 1390.40 | 306.18 | 38.42 | 3684.93 | 21.24 | 3.28 | 176.41 | 104.92 | | 314.5 |
| REC37 | 7.80 | 6.04 | 15.43 | 16.49 | 0.53 | 41.78 | | 4.35 | 8.93 | 32.41 | 0.12 | <1.28 | | 37822.47 | 1419.55 | 16.57 | 18767.63 | 29.96 | <1.6 | 0.30 | 427.31 | | 195.79 |
| REC38 | 14.82 | 39.20 | 33.32 | 20.13 | 0.80 | 11.61 | | 2.52 | 5.72 | 30.38 | 0.51 | 4.16 | | 4978.63 | 451.01 | 26.59 | 3774.61 | 8.82 | 0.62 | 161.33 | 355.52 | | 233.80 |
| REC39 | 6.27 | 16.28 | 59.78 | 65.68 | <1.28 | 65.13 | | 2.22 | 4.58 | 106.00 | <3.2 | 1.05 | | 1497.35 | 1760.53 | 21.71 | 17594.05 | 41.09 | <1.6 | 76.23 | 508.20 | | 335.7 |
| REC40 | 80.40 | 73.38 | 55.83 | 11.57 | 2.86 | 2.34 | | 3.41 | 3.65 | 47.28 | 7.23 | 13.01 | | 1080.89 | 229.80 | 40.64 | 3973.34 | 26.66 | 7.77 | 90.99 | 209.67 | | 117.0 |
| **Mean** | 16.68 | 52.49 | 53.97 | 15.74 | 2.60 | 21.50 | | 5.83 | 11.41 | 60.21 | 5.77 | 13.03 | | 5408.34 | 890.59 | 42.29 | 5994.50 | 30.90 | 6.38 | 72.97 | 304.36 | | 252.95 |
| **SD** | 18.17 | 37.82 | 55.63 | 13.26 | 1.77 | 20.85 | | 9.15 | 13.14 | 67.78 | 5.09 | 16.04 | | 11256.48 | 1313.74 | 31.61 | 5882.80 | 19.78 | 6.50 | 93.08 | 238.76 | | 141.70 |
| **Median** | 13.48 | 51.53 | 44.22 | 12.58 | 2.04 | 11.43 | | 2.52 | 6.67 | 42.08 | 4.49 | 11.39 | | 1390.40 | 508.60 | 38.42 | 3851.95 | 28.22 | 3.28 | 33.16 | 256.18 | | 233.80 |
| **IQR** | 9.07 | 56.82 | 48.78 | 11.39 | 1.92 | 27.03 | | 3.74 | 6.54 | 35.04 | 6.19 | 15.31 | | 1604.04 | 598.99 | 16.23 | 1629.52 | 18.35 | 8.15 | 123.60 | 337.65 | | 200.57 |
| **% Elevated** | 8.7 | 43.5 | 65.2 | 4.4 | 60.9 | 95.7 | | 82.6 | 100.0 | 47.8 | 52.2 | 26.1 | | 95.7 | 65.2 | 60.9 | 26.1 | 39.1 | 34.8 | 34.8 | 100.0 | | 13.0 |
| **Control Mean + 2xSD** | 33.40 | 50.57 | 25.90 | 37.00 | 1.28 | 0.87 | | 0.64 | 2.37 | 46.10 | 3.68 | 14.88 | | 144.98 | 338.62 | 31.57 | 4816.80 | 31.96 | 8.28 | 54.93 | 8.10 | | 380.66 |
|  |  |  |  |  |  |  | |  |  |  |  |  | |  |  |  |  |  |  |  |  | |  |
| **TRACK 3** | | | | | | | | | | | | | | | | | | | | | | | |
| REC02 | 7.93 | 10.44 | 3.76 | 6.63 | <1.28 | 36.76 | | <0.64 | 2.12 | 5.13 | 0.31 | 1.69 | | 353.85 | 357.41 | 15.89 | 3726.93 | 10.90 | <1.6 | 58.16 | 8.33 | | 811.4 |
| REC04 | 27.36 | 32.77 | 15.34 | 14.72 | 1.14 | 7.13 | | 1.26 | 5.59 | 16.89 | 3.73 | 9.72 | | 218.44 | 303.59 | 28.67 | 2772.75 | 17.78 | 5.72 | 107.79 | 40.43 | | 297.9 |
| REC12 | 62.91 | 8.07 | 12.46 | 4.83 | <1.28 | 31.57 | | 14.44 | 28.21 | 7.70 | 0.89 | 1.05 | | 35608.28 | 1832.77 | 92.26 | 11402.95 | 29.38 | 4.31 | 342.00 | 452.40 | | 343.0 |
| REC14 | 8.65 | 39.18 | 8.11 | 6.94 | 0.58 | 1.49 | | <0.64 | 3.40 | 17.28 | 2.22 | 6.66 | | 134.54 | 312.41 | 26.09 | 2410.40 | 11.28 | 1.61 | 37.68 | 144.44 | | 158.3 |
| REC18 | 13.63 | 11.51 | <1.28 | 5.09 | <1.28 | 67.43 | | 0.64 | 8.46 | <6.4 | <3.2 | 1.49 | | 295.55 | 379.96 | 27.44 | 3188.09 | 13.18 | <1.6 | 2.86 | 20.05 | | 343.7 |
| REC19 | <3.2 | 1.39 | <1.28 | 2.50 | <1.28 | 7.51 | | 1.36 | 19.73 | 7.70 | 3.2 | <1.28 | | 680.33 | 3454.87 | 34.38 | 12279.94 | 4.53 | <1.6 | 46.45 | 227.10 | | 210.3 |
| REC21 | 34.20 | 32.61 | <1.28 | 4.32 | 0.87 | 4.73 | | 1.57 | 5.21 | 10.22 | 1.65 | 9.43 | | 1670.64 | 140.87 | 43.06 | 5107.61 | 16.82 | <1.6 | 102.28 | 165.74 | | 269.9 |
| REC23 | 12.93 | 50.41 | 38.39 | 25.41 | 1.19 | 57.48 | | 3.79 | 25.69 | 33.63 | 4.87 | 14.91 | | 546.84 | 2072.27 | 43.17 | 13120.73 | 22.01 | 3.15 | 99.33 | 76.40 | | 134.4 |
| REC26 | 13.55 | 8.02 | 4.22 | 4.32 | <1.28 | 117.47 | | 1.74 | 11.63 | 2.53 | <3.2 | 2.49 | | 1364.48 | 1299.47 | 54.04 | 5622.63 | 10.90 | <1.6 | 97.91 | 31.27 | | 443.2 |
| REC29 | 23.56 | 13.13 | 7.07 | 9.36 | <1.28 | 37.80 | | 4.59 | 12.77 | 35.24 | 0.89 | 4.74 | | 2107.27 | 437.43 | 20.94 | 5942.81 | 19.31 | 16.98 | 603.16 | 1358.15 | | 270.6 |
| REC34 | <3.2 | <8 | <1.28 | 0.91 | <1.28 | 17.87 | | 0.29 | 43.16 | <6.4 | <3.2 | <1.28 | | 479.38 | 543.91 | 49.08 | 11570.78 | 13.18 | <1.6 | 1.38 | 134.15 | | 108.5 |
| REC36 | 13.63 | <8 | 92.90 | 15.28 | <1.28 | 85.17 | | 17.02 | 31.94 | <6.4 | <3.2 | <1.28 | | 865.59 | 814.42 | 12.38 | 4860.47 | 10.52 | <1.6 | 143.65 | 638.58 | | 2226.28 |
| **Mean** | 18.73 | 18.63 | 15.61 | 8.36 | 1.17 | 39.37 | | 4.00 | 16.49 | 12.96 | 2.55 | 4.67 | | 3693.77 | 995.78 | 37.28 | 6833.84 | 14.98 | 3.58 | 136.89 | 274.75 | | 468.13 |
| **SD** | 16.79 | 15.73 | 26.50 | 6.95 | 0.22 | 36.35 | | 5.66 | 13.17 | 10.95 | 1.36 | 4.55 | | 10069.47 | 1000.51 | 21.66 | 4052.37 | 6.53 | 4.43 | 171.90 | 390.57 | | 583.97 |
| **Median** | 13.59 | 10.97 | 5.65 | 5.86 | 1.28 | 34.16 | | 1.46 | 12.20 | 7.70 | 3.20 | 2.09 | | 613.59 | 490.67 | 31.52 | 5365.12 | 13.18 | 1.60 | 98.62 | 139.30 | | 284.23 |
| **IQR** | 16.04 | 24.64 | 11.90 | 6.38 | 0.10 | 52.55 | | 3.35 | 20.83 | 10.59 | 1.74 | 6.08 | | 1101.74 | 1086.64 | 19.84 | 7852.69 | 7.26 | 1.84 | 72.49 | 245.29 | | 171.30 |
| **% Elevated** | 16.67 | 0.00 | 16.67 | 0.00 | 0.00 | 100.00 | | 75.00 | 91.67 | 0.00 | 8.33 | 8.33 | | 91.67 | 75.00 | 50.00 | 66.67 | 0.00 | 8.33 | 66.67 | 100.00 | | 25.00 |
| P value^ | 0.37 | 0.0004 | 0.005 | 0.019 | 0.0004 | 0.068 | | 0.24 | 0.14 | 0.002 | 0.004 | 0.013 | | 0.33 | 0.40 | 0.29 | 0.31 | 0.001 | 0.072 | 0.14 | 0.41 | | 0.12 |
| ^δ^ Concentration values highlighted in gray are considered elevated above the normal control mean + 2xSD; unit is pg/ml unless otherwise noted  ^T-test was used to compare Means of Track 2 and Track 3  SD, Standard Deviation of the Mean; IQR, Interquartile Range  Median, IQR, Mean and SD were calculated using the lowest value detected for any value listed as (<). | | | | | | | | | | | | | | | | | |  | | | |  |  |
