# Supplementary material for: Elevated cytokines and chemokines in peripheral blood of patients with SARS-CoV-2 pneumonia treated with high-titer convalescent plasma
Source: PLoS Pathog. 2021 Oct 29;17(10):e1010025. doi: 10.1371/journal.ppat.1010025 (PMC8580259; doi:10.1371/journal.ppat.1010025)
Supplement: S8 Table — (DOCX) [file ppat.1010025.s009.docx]

**S8 Table. Day 3 Individual Recipient Cytokine Levels of Interest**

| **Recipients** | **EGF** | **IFNα2** | **IFNβ** | **IL-1RA** | **IL-3** | **IL-6** | **IL-7** | **IL-8 (CXCL8)** | **IL-12p40** | **IL-12p70** | **IL-17A** | **IP-10 (CXCL10)** | **MCP-1 (CCL2)** | **MIP-1β (CCL4)** | **RANTES (CCL5)** | **TNFα** | **TNFβ** | **VEGF** | **CRP ug/ml** | **NGAL ng/ml** |
| --- | --- | --- | --- | --- | --- | --- | --- | --- | --- | --- | --- | --- | --- | --- | --- | --- | --- | --- | --- | --- |
| **TRACK 2** | | | | | | | | | | | | | | | | | | | | |
| REC01 | 4.91 | 37.87 | 4.73 | 7.23 | 0.15 | 46.30 | 1.88 | 3.95 | 20.44 | 3.54 | 9.60 | 219.57 | 278.02 | 28.38 | 5398.93 | 22.83 | 2.01 | <2.56 | 358.80 | 354.70 |
| REC03 | 6.65 | 21.53 | 3.49 | 6.82 | <1.28 | 1.12 | 0.13 | 7.35 | 43.96 | 2.04 | 3.94 | 209.22 | 676.30 | 29.99 | 7083.55 | 23.04 | <1.6 | 19.36 | 4.97 | 237.76 |
| REC05 | 9.31 | 52.76 | 5.49 | 6.82 | 0.29 | 2.04 | 49.12 | 2.04 | 23.70 | 10.17 | 9.29 | 214.71 | 303.82 | 37.86 | 3112.97 | 25.36 | 2.03 | 5.31 | 25.05 | 71.54 |
| REC06 | 13.19 | 69.33 | 53.36 | 21.58 | 0.66 | 7.44 | 17.75 | 3.82 | 58.43 | 8.21 | 59.54 | 476.55 | 561.62 | 38.63 | 4286.34 | 27.47 | 2.46 | 30.61 | 273.88 | 122.53 |
| REC07 | <3.2 | 1.67 | <1.28 | 3.63 | <1.28 | 7.02 | <0.64 | 3.05 | <6.4 | <3.2 | <1.28 | 344.33 | 755.93 | 29.27 | 1501.91 | 14.38 | <1.6 | 6.45 | 62.83 | 130.62 |
| REC08 | 52.23 | 18.23 | 1.77 | 188.3 | <1.28 | 163.5 | 4.20 | 75.04 | 8.13 | <3.2 | 1.39 | 35687.7 | 6690.59 | 184.66 | 6847.30 | 99.03 | 5.99 | 144.58 | 934.36 | 849.16 |
| REC09 | <3.2 | 40.49 | 17.71 | 9.43 | 0.26 | 32.72 | 0.41 | 9.24 | 30.21 | 2.60 | 5.99 | 939.18 | 431.01 | 27.19 | 4981.25 | 22.83 | <1.6 | 15.03 | 547.99 | 75.71 |
| REC10 | 7.09 | 20.92 | 3.49 | 3.05 | <1.28 | 1276.1 | <0.64 | 32.85 | 96.77 | 0.70 | 1.39 | 2374.25 | 871.87 | 23.60 | 3340.43 | 77.15 | 3.82 | 15.75 | 248.69 | 272.39 |
| REC11 | <3.2 | <8 | <1.28 | 5.55 | 1.85 | 603.6 | 0.61 | 87.94 | 8.49 | 4.11 | <1.28 | 586.19 | 41880.2 | 22.13 | 912.62 | 16.92 | <1.6 | 4.58 | 237.50 | 69.34 |
| REC13 | 26.88 | 151.1 | 28.29 | 16.45 | 6.46 | 6.94 | 3.65 | 6.22 | 148.9 | 25.68 | 33.45 | 471.48 | 439.84 | 58.31 | 3289.84 | 90.19 | 22.85 | 52.98 | 252.38 | 215.90 |
| REC15 | 50.97 | 40.97 | 5.04 | 12.10 | 0.54 | 6.35 | 1.20 | 7.26 | 15.73 | 2.23 | 8.32 | 436.18 | 447.29 | 36.50 | 5049.38 | 22.62 | 5.41 | 106.13 | 157.28 | 727.51 |
| REC16 | 87.37 | 34.59 | 6.24 | 23.63 | 0.48 | 2.25 | 2.29 | 7.53 | 18.63 | 5.04 | 10.25 | 103.77 | 594.08 | 59.08 | 4435.97 | 34.63 | 14.04 | 399.76 | 8.70 | 784.53 |
| REC17 | 45.52 | 27.41 | 6.82 | 8.91 | <1.28 | 2.28 | 9.14 | 18.21 | 13.56 | 2.60 | 6.67 | 292.32 | 414.54 | 30.70 | 6155.78 | 27.26 | 1.35 | 140.60 | 83.98 | 322.89 |
| REC22 | <3.2 | <8 | <1.28 | 9.14 | <1.28 | 55.74 | 2.39 | 16.41 | 3.42 | <3.2 | <1.28 | 2609.23 | 4117.00 | 36.16 | 5504.36 | 15.86 | <1.6 | 9.34 | 1589.72 | 108.52 |
| REC24 | 8.58 | 9.85 | <1.28 | 3.92 | <1.28 | 12.36 | 2.29 | 8.75 | 30.57 | 0.70 | 3.41 | 286.05 | 420.18 | 29.64 | 6134.97 | 20.93 | 2.46 | 46.91 | 101.06 | 127.65 |
| REC25 | 39.72 | 124.7 | 25.08 | 15.29 | 3.56 | 4.23 | 3.73 | 7.25 | 81.22 | 19.92 | 34.19 | 248.85 | 185.27 | 46.99 | 3418.57 | 57.37 | 24.53 | 130.51 | 23.78 | 276.11 |
| REC27 | 4.08 | 34.68 | <1.28 | 3.92 | <1.28 | 0.58 | 0.74 | 6.52 | 11.03 | 2.04 | 5.15 | 44.15 | 134.73 | 41.14 | 5539.25 | 8.03 | <1.6 | <2.56 | 310.70 | 244.98 |
| REC33 | 6.18 | 27.86 | 13.91 | 6.71 | <1.28 | 11.35 | <0.64 | 10.17 | 41.43 | 2.70 | 2.87 | 3220.37 | 481.51 | 25.12 | 3011.55 | 31.90 | <1.6 | <2.56 | 899.13 | 121.70 |
| REC35 | 28.91 | 51.87 | 7.12 | 15.47 | 0.95 | 3.29 | 2.00 | 5.12 | 26.59 | 4.20 | 13.40 | 818.76 | 195.32 | 39.66 | 3315.02 | 30.00 | 10.74 | 184.77 | 249.45 | 323.01 |
| REC37 | 3.17 | 15.83 | 2.20 | 5.31 | <1.28 | 35.76 | 1.09 | 7.37 | 13.56 | 1.28 | 3.23 | 1466.37 | 1182.99 | 31.35 | 6523.01 | 40.95 | <1.6 | 25.96 | 990.92 | 243.69 |
| REC38 | 26.55 | 26.82 | 9.15 | 16.22 | <1.28 | 18.50 | 4.51 | 12.61 | 37.45 | 3.36 | 7.33 | 16763.4 | 477.42 | 33.75 | 4873.27 | 22.83 | 8.99 | 464.61 | 392.36 | 226.55 |
| REC39 | 21.02 | 24.51 | 2.21 | 24.74 | <1.28 | 36.01 | 0.73 | 7.63 | 51.92 | 0.71 | 6.00 | 304.57 | 689.77 | 30.87 | 4208.31 | 36.32 | 10.74 | 233.07 | 460.81 | 615.79 |
| REC40 | 133.4 | 15.83 | <1.28 | 76.01 | <1.28 | 2.01 | 1.40 | 7.79 | 7.04 | <3.2 | 1.36 | 248.41 | 308.03 | 43.01 | 3881.90 | 11.42 | 0.70 | 218.41 | 124.21 | 830.74 |
| **Mean** | 25.59 | 37.60 | 8.86 | 21.31 | 1.38 | 101.6 | 4.83 | 15.40 | 35.96 | 4.98 | 10.03 | 2972.42 | 2719.01 | 41.91 | 4469.85 | 33.88 | 5.69 | 98.37 | 362.55 | 319.71 |
| **SD** | 32.06 | 35.74 | 12.29 | 39.38 | 1.30 | 285.4 | 10.38 | 21.90 | 34.95 | 6.10 | 13.99 | 7918.82 | 8664.70 | 32.58 | 1611.57 | 24.32 | 6.78 | 128.97 | 395.50 | 255.27 |
| **Median** | 9.31 | 27.41 | 4.73 | 9.14 | 1.28 | 7.44 | 1.88 | 7.53 | 23.70 | 3.20 | 5.99 | 436.18 | 477.42 | 33.75 | 4435.97 | 25.36 | 2.03 | 30.61 | 249.45 | 243.69 |
| **IQR** | 29.82 | 23.70 | 6.61 | 10.21 | 0.48 | 33.10 | 3.00 | 5.02 | 30.40 | 2.02 | 7.32 | 954.14 | 361.56 | 10.94 | 2194.08 | 13.70 | 5.89 | 134.69 | 334.06 | 213.77 |
| **% Elevated** | 26.1 | 21.7 | 8.7 | 8.7 | 13.0 | 95.7 | 69.9 | 95.7 | 21 | 7 | 13.0 | 91.3 | 73.9 | 52.2 | 47.8 | 30.4 | 26.1 | 39.1 | 95.7 | 13.0 |
| **Control Mean + 2xSD** | 33.40 | 50.57 | 25.90 | 37.00 | 1.28 | 0.87 | 0.64 | 2.37 | 46.1 | 3.68 | 14.88 | 144.98 | 338.62 | 31.57 | 4816.80 | 31.96 | 8.28 | 54.93 | 8.10 | 380.66 |
|  |  |  |  |  |  |  |  |  |  |  |  |  |  |  |  |  |  |  |  |  |
| **TRACK 3** | | | | | | | | | | | | | | | | | | | | |
| REC02 | 8.58 | 17.21 | 3.80 | 2.82 | <1.28 | 4.39 | <0.64 | 1.14 | 7.77 | 0.41 | 1.76 | 33.33 | 152.08 | 14.54 | 3801.48 | 6.76 | <1.6 | 11.53 | 0.56 | 252.17 |
| REC04 | <3.2 | 15.14 | 1.54 | 31.49 | <1.28 | 40.55 | 0.10 | 2.48 | 4.87 | <3.2 | 1.76 | 411.54 | 623.79 | 23.99 | 4196.47 | 16.71 | <1.6 | 23.98 | 569.30 | 228.13 |
| REC12 | 5.57 | 52.92 | 9.86 | 85.31 | 0.72 | 28.37 | 0.49 | 6.96 | 25.15 | 6.35 | 12.46 | 546.29 | 539.26 | 51.39 | 4472.62 | 34.63 | 4.49 | 53.60 | 726.50 | 212.95 |
| REC14 | 31.13 | 20.90 | 2.20 | 9.25 | <1.28 | 4.44 | 1.72 | 25.10 | 7.77 | 0.70 | 5.32 | 170.05 | 187.83 | 31.03 | 2522.46 | 18.82 | 5.29 | 322.02 | 8.65 | 184.56 |
| REC18 | 9.79 | 9.04 | 14.73 | 281.0 | <1.28 | 7857.4 | 0.57 | 383.1 | 15.37 | 0.32 | 1.76 | 1012.78 | 6642.23 | 488.89 | 4855.06 | 276.76 | 21.41 | 14.66 | 96.97 | 457.48 |
| REC19 | 37.85 | 17.21 | <1.28 | 22.05 | <1.28 | 5.60 | 1.16 | 14.21 | 7.04 | <3.2 | 2.14 | 1029.87 | 1440.70 | 54.59 | 4204.55 | 16.92 | <1.6 | 99.45 | 595.95 | 365.22 |
| REC21 | 27.34 | 13.29 | <1.28 | 10.01 | <1.28 | 9.21 | <0.64 | 15.06 | 4.50 | <3.2 | 2.88 | 852.57 | 286.68 | 72.85 | 5005.36 | 18.82 | <1.6 | 80.20 | 33.82 | 722.00 |
| REC23 | 59.59 | 59.24 | 10.43 | 22.81 | 1.48 | 6.72 | 4.20 | 9.10 | 25.51 | 4.39 | 15.87 | 310.19 | 709.08 | 41.31 | 3068.82 | 24.94 | 7.60 | 245.72 | 204.03 | 351.49 |
| REC26 | 27.19 | 31.31 | 2.36 | 9.72 | <1.28 | 47.75 | 0.59 | 4.50 | 20.08 | <3.2 | 6.66 | 599.05 | 283.82 | 51.72 | 3067.73 | 17.97 | <1.6 | 55.80 | 8.42 | 238.52 |
| REC29 | 34.00 | 19.67 | 2.20 | 13.78 | <1.28 | 12.82 | <0.64 | 12.88 | 17.18 | <3.2 | 5.99 | 17302.9 | 941.43 | 35.09 | 5025.05 | 31.26 | 13.80 | 237.12 | 1084.16 | 888.91 |
| REC34 | 12.11 | <8 | 10.57 | 337.3 | <1.28 | 9496.6 | 1.83 | 2260.0 | 16.10 | <3.2 | <1.28 | 1089.71 | 5202.28 | 445.39 | 1967.00 | 564.52 | 23.70 | <2.56 | 2216.15 | 627.05 |
| REC36 | <3.2 | 7.63 | <1.28 | 65.87 | <1.28 | 350.4 | <0.64 | 10.16 | 4.50 | <3.2 | 0.15 | 3035.26 | 802.85 | 19.74 | 3925.09 | 42.01 | <1.6 | 15.84 | 221.76 | 313.03 |
| **Mean** | 21.63 | 22.63 | 5.13 | 74.28 | 1.25 | 1488.7 | 1.10 | 228.7 | 12.99 | 2.88 | 4.84 | 2199.46 | 1484.34 | 110.88 | 3842.64 | 89.18 | 7.16 | 96.87 | 480.52 | 403.46 |
| **SD** | 17.45 | 16.98 | 4.83 | 113.1 | 0.18 | 3377.2 | 1.10 | 648.6 | 7.91 | 1.72 | 4.85 | 4819.52 | 2126.76 | 167.48 | 995.29 | 166.80 | 8.07 | 109.20 | 648.39 | 583.97 |
| **Median** | 19.65 | 17.21 | 2.28 | 22.43 | 1.28 | 20.59 | 0.64 | 11.52 | 11.57 | 3.2 | 2.51 | 725.81 | 666.43 | 46.35 | 4060.78 | 21.88 | 3.05 | 54.70 | 212.90 | 332.23 |
| **IQR** | 24.02 | 11.27 | 8.53 | 60.79 | 0.00 | 116.98 | 0.71 | 11.22 | 11.41 | 0.62 | 4.40 | 658.63 | 780.28 | 29.89 | 1499.68 | 18.77 | 7.55 | 118.32 | 601.06 | 263.95 |
| **% Elevated** | 25.0 | 16.7 | 0.0 | 33.3 | 8.3 | 100.0 | 33.3 | 91.7 | 0.0 | 16.7 | 8.3 | 91.7 | 66.7 | 66.7 | 25.0 | 33.3 | 25.0 | 50.0 | 91.7 | 33.3 |
| **P value^** | 0.320 | 0.051 | 0.105 | 0.070 | 0.296 | 0.092 | 0.051 | 0.139 | 0.003 | 0.067 | 0.060 | 0.361 | 0.262 | 0.092 | 0.083 | 0.139 | 0.298 | 0.486 | 0.286 | 0.165 |
| ^δ^ Concentration values highlighted in gray are considered elevated above the normal control mean + 2xSD; unit is pg/ml unless otherwise noted  ^T-test was used to compare Means of Track 2 and Track 3  SD, Standard Deviation of the Mean; IQR, Interquartile Range  Median, IQR, Mean and SD were calculated using the lowest value detected for any value listed as (<). | | | | | | | | | | | | | | | | | | | | |
