# Supplementary material for: Elevated cytokines and chemokines in peripheral blood of patients with SARS-CoV-2 pneumonia treated with high-titer convalescent plasma
Source: PLoS Pathog. 2021 Oct 29;17(10):e1010025. doi: 10.1371/journal.ppat.1010025 (PMC8580259; doi:10.1371/journal.ppat.1010025)
Supplement: S9 Table — (DOCX) [file ppat.1010025.s010.docx]

**S9 Table. Day 10 Individual Recipient Cytokine Levels of Interest**

| **Recipient** | **EGF** | **IFNα2** | **IFNγ** | **IL-1RA** | **IL-3** | **IL-6** | **IL-7** | **IL-8 (CXCL8)** | **IL-12p40** | **IL-12p70** | **IL-17A** | **IP-10 (CXCL10)** | **MCP-1 (CCL2)** | **MIP-1β (CCL4)** | **RANTES (CCL5)** | **TNFα** | **TNFβ** | **VEGF** | **CRP ug/ml** | **NGAL ng/ml** |
| --- | --- | --- | --- | --- | --- | --- | --- | --- | --- | --- | --- | --- | --- | --- | --- | --- | --- | --- | --- | --- |
| **TRACK 2** | | | | | | | | | | | | | | | | | | | | |
| REC01 | 2.18 | 27.08 | 6.98 | 7.57 | <1.28 | 1.50 | 5.03 | 8.34 | 7.36 | 0.75 | 7.41 | 302.39 | 170.45 | 34.84 | 4748.70 | 22.02 | 4.54 | <2.56 | 30.22 | 927.5 |
| REC03 * | - | - | - | - | - | - | - | - | - | - | - | - | - | - | - | - | - | - | - | - |
| REC05 | 38.08 | 34.74 | 17.11 | 65.58 | 0.10 | 1.20 | 57.58 | 5.09 | 22.13 | 5.45 | 10.94 | 384.18 | 547.52 | 48.66 | 4132.60 | 25.56 | 3.19 | 139.76 | 11.86 | 656.6 |
| REC06 | <3.2 | 9.25 | 24.11 | 18.24 | <1.28 | 0.89 | 141.35 | 6.56 | 70.59 | <3.2 | 8.77 | 632.42 | 941.61 | 33.77 | 6677.48 | 30.21 | 2.69 | 853.21 | 6.21 | 339.4 |
| REC07 | 15.00 | 10.20 | <1.28 | 61.29 | <1.28 | 4.19 | <0.64 | 12.77 | <6.4 | <3.2 | 3.10 | 143.08 | 158.37 | 62.79 | 4803.64 | 12.16 | <2.56 | 213.06 | 1.24 | 2305.5 |
| REC08 | 22.62 | 0.82 | 8.91 | 3985.12 | 0.06 | 681.69 | 6.48 | 63.37 | 13.24 | <3.2 | <1.28 | 24502.5 | 1508.58 | 218.53 | 5142.29 | 321.88 | 6.65 | 428.20 | 1357.7 | 3127.5 |
| REC09 | 11.12 | 11.76 | 8.05 | 10.01 | <1.28 | 11.64 | <0.64 | 7.27 | 1.76 | <3.2 | 0.89 | 559.02 | 251.85 | 34.11 | 3098.53 | 12.16 | <2.56 | 109.89 | 90.27 | 470.8 |
| REC10 | 12.84 | 5.97 | 5.78 | 4.41 | 0.72 | 922.29 | 1.62 | 57.92 | 20.59 | <3.2 | 2.05 | 5556.84 | 2588.50 | 77.97 | 3880.25 | 145.46 | 3.80 | 73.07 | 149.76 | 314.7 |
| REC11 | 6.10 | 10.20 | <1.28 | 12.01 | <1.28 | 11.83 | <0.64 | 20.16 | 6.58 | <3.2 | 2.84 | 1863.85 | 1108.83 | 39.13 | 3064.34 | 19.90 | <2.56 | 77.86 | 289.95 | 423.2 |
| REC13 | 65.93 | 32.86 | 8.73 | 93.87 | <1.28 | 1.92 | <0.64 | 2.00 | 102.75 | <3.2 | 7.34 | 500.58 | 475.88 | 37.81 | 2771.47 | 33.76 | 4.68 | 390.50 | 14.23 | 1741.5 |
| REC15 | 85.55 | 29.98 | <1.28 | 142.38 | <1.28 | 2.45 | <0.64 | 7.30 | 20.58 | <3.2 | <1.28 | 589.30 | 629.50 | 43.44 | 4766.65 | 13.91 | 2.20 | 138.52 | 65.94 | 2479.8 |
| REC16 | 133.09 | 18.82 | <1.28 | 163.14 | <1.28 | 1.20 | 0.44 | 4.59 | 25.20 | 0.75 | 6.94 | 142.76 | 291.49 | 44.52 | 2669.72 | 14.26 | 3.80 | 314.17 | 13.96 | 1461.8 |
| REC17 | 79.04 | 6.10 | <1.28 | 343.23 | <1.28 | 3.99 | 7.34 | 14.14 | 16.34 | <3.2 | 0.27 | 573.44 | 290.76 | 53.20 | 3107.68 | 19.54 | 3.06 | 466.48 | 172.24 | 4192.6 |
| REC22 | 2.69 | 5.22 | 11.38 | 134.33 | <1.28 | 1501.16 | 1.28 | 158.35 | 13.62 | <3.2 | 2.30 | 2622.88 | 2893.75 | 166.96 | 3111.75 | 68.47 | 4.05 | 4.78 | 83.47 | 661.5 |
| REC24 | 77.96 | 5.22 | <1.28 | 18.40 | <1.28 | 1.02 | <0.64 | 6.58 | 25.97 | <3.2 | 0.27 | 403.79 | 271.68 | 27.77 | 2167.53 | 12.51 | <2.56 | 68.51 | 4.44 | 349.4 |
| REC25 | 86.47 | 60.70 | 15.45 | 20.64 | 2.03 | 1.44 | 0.18 | 2.77 | 42.33 | 4.59 | 19.77 | 358.52 | 228.49 | 30.36 | 2145.97 | 18.66 | 5.04 | 121.50 | 2.11 | 858.7 |
| REC27 | 39.90 | 17.26 | <1.28 | 101.73 | <1.28 | 0.32 | <0.64 | 3.51 | 11.29 | <3.2 | 3.59 | 278.68 | 307.00 | 46.67 | 2871.28 | 1.80 | <2.56 | 24.05 | 6.18 | 2290.2 |
| REC33 | 31.35 | 13.22 | <1.28 | 50.46 | <1.28 | 1.63 | <0.64 | 3.10 | 3.38 | <3.2 | 1.43 | 106.73 | 206.69 | 22.83 | 2374.25 | 13.56 | <2.56 | 35.86 | 19.26 | 567.2 |
| REC35 | 37.63 | 35.95 | <1.28 | 121.77 | <1.28 | 1.41 | 0.60 | 4.02 | 23.66 | 1.29 | 8.53 | 282.12 | 203.82 | 35.41 | 2404.96 | 22.19 | 11.00 | 192.98 | 7.00 | 2988.4 |
| REC37 | 2.18 | 4.83 | <1.28 | 10.73 | <1.28 | 263.79 | <0.64 | 4.19 | 8.94 | <3.2 | 0.26 | 517.45 | 793.84 | 40.68 | 1859.95 | 33.39 | <2.56 | <2.56 | 78.95 | 2141.6 |
| REC38 | 18.57 | 14.53 | <1.28 | 55.42 | <1.28 | 5.89 | <0.64 | 4.28 | 1.37 | <3.2 | 0.89 | 309.98 | 388.93 | 26.93 | 2469.70 | 4.35 | <2.56 | 183.73 | 14.21 | 1465.9 |
| REC39 | 10.40 | 60.60 | 16.28 | 18.51 | 0.87 | 7.19 | 0.18 | 4.09 | 41.97 | 4.76 | 20.44 | 84.38 | 447.92 | 42.11 | 1741.10 | 28.58 | 10.75 | 195.16 | 112.67 | 2184.2 |
| REC40 | 51.15 | 6.93 | <1.28 | 36.67 | 0.13 | 4.75 | 5.78 | 4.70 | 14.79 | <3.2 | <1.28 | 278.42 | 600.23 | 38.62 | 2699.47 | 13.39 | <2.56 | 140.79 | 21.15 | 3306.4 |
| **Mean** | 37.86 | 19.19 | 6.28 | 248.89 | 1.10 | 156.06 | 10.65 | 18.41 | 22.76 | 3.13 | 5.09 | 1863.33 | 695.71 | 54.87 | 3304.97 | 40.35 | 4.02 | 208.60 | 116.05 | 1602.48 |
| **SD** | 36.01 | 17.09 | 6.77 | 838.14 | 0.48 | 384.40 | 31.60 | 35.31 | 24.01 | 1.09 | 5.83 | 5200.96 | 745.60 | 46.93 | 1257.30 | 69.56 | 2.47 | 201.00 | 286.54 | 1147.21 |
| **Median** | 26.98 | 12.49 | 1.28 | 52.94 | 1.28 | 3.22 | 0.64 | 5.82 | 16.34 | 3.20 | 2.57 | 393.99 | 418.43 | 39.91 | 2967.81 | 19.72 | 2.88 | 139.14 | 20.21 | 1463.85 |
| **IQR** | 51.66 | 22.95 | 7.58 | 98.48 | 0.00 | 9.11 | 3.54 | 7.55 | 16.26 | 0.00 | 6.11 | 305.80 | 495.94 | 13.87 | 1648.37 | 16.37 | 1.86 | 138.94 | 80.35 | 1712.12 |
| **% Elevated** | 45.5 | 9.1 | 0.0 | 54.6 | 4.6 | 95.5 | 36.4 | 95.5 | 9.1 | 13.6 | 9.1 | 81.8 | 54.6 | 81.8 | 9.1 | 22.7 | 9.1 | 77.3 | 72.7 | 86.4 |
| **Control Mean + 2xSD** | 33.40 | 50.57 | 25.90 | 37.00 | 1.28 | 0.87 | 0.64 | 2.37 | 46.10 | 3.68 | 14.88 | 144.98 | 338.62 | 31.57 | 4816.80 | 31.96 | 8.28 | 54.93 | 8.10 | 380.66 |
|  |  |  |  |  |  |  |  |  |  |  |  |  |  |  |  |  |  |  |  |  |
| **TRACK 3** | | | | | | | | | | | | | | | | | | | | |
| REC02 | 15.14 | 6.19 | 87.87 | 56.67 | 0.37 | 13.91 | <0.64 | 12.49 | 9.72 | 1.20 | 0.27 | 223.02 | 1041.18 | 24.21 | 5755.71 | 26.80 | <2.56 | 582.45 | 37.69 | 1334.01 |
| REC04 | 51.17 | 39.22 | 12.18 | 28.06 | 0.66 | 8.45 | 1.68 | 5.62 | 15.18 | 2.86 | 13.51 | 439.64 | 465.21 | 31.78 | 2950.69 | 25.38 | 10.00 | 664.70 | 163.43 | 575.42 |
| REC12 * | - | - | - | - | - | - | - | - | - | - | - | - | - | - | - | - | - | - | - | - |
| REC14 | 19.02 | 6.93 | <1.28 | 81.44 | <1.28 | 1.15 | <0.64 | 6.77 | 32.85 | <3.2 | 3.59 | 599.53 | 494.29 | 35.41 | 1788.05 | 19.72 | <2.56 | 156.56 | 5.04 | 1016.42 |
| REC18 | <3.2 | 3.12 | 22.52 | 308.19 | <1.28 | 3377.42 | 2.12 | 598.66 | 4.18 | <3.2 | <1.28 | 239.60 | 3759.13 | 63.81 | 3035.72 | 145.46 | 13.98 | 8.02 | 672.39 | 2312.67 |
| REC19 | 13.93 | <8 | 84.34 | 93.33 | <1.28 | 1101.30 | <0.64 | 41.19 | 12.85 | <3.2 | <1.28 | 2569.14 | 3026.20 | 164.13 | 3054.79 | 71.36 | 3.55 | 96.84 | 98.51 | 840.22 |
| REC21 | 30.40 | <8 | 3.49 | 71.07 | <1.28 | 2746.72 | <0.64 | 16.39 | <6.4 | <3.2 | <1.28 | 862.52 | 348.48 | 67.08 | 3515.53 | 103.59 | 14.35 | 112.15 | 348.82 | 6187.99 |
| REC23 | 14.54 | 27.75 | 8.91 | 34.62 | <1.28 | 63.89 | <0.64 | 7.80 | 19.04 | 2.17 | 11.17 | 578.46 | 449.99 | 33.06 | 2498.79 | 14.44 | 3.56 | 225.88 | 7.83 | 865.02 |
| REC26 | 23.39 | 42.98 | 15.45 | 28.50 | 0.51 | 2.55 | 4.92 | 10.53 | 14.37 | 2.51 | 14.70 | 279.08 | 182.03 | 45.55 | 2190.56 | 17.08 | 6.40 | 424.20 | 91.28 | 4092.86 |
| REC29 | 25.21 | 3.12 | 1.44 | 62.76 | <1.28 | 55.76 | <0.64 | 4.72 | 25.20 | <3.2 | 4.10 | 546.08 | 536.01 | 31.44 | 2553.83 | 28.22 | 10.50 | 146.87 | 20.83 | 1010.57 |
| REC34 * | - | - | - | - | - | - | - | - | - | - | - | - | - | - | - | - | - | - | - | - |
| REC36 | 19.84 | <8 | <1.28 | 118.24 | <1.28 | 59.96 | <0.64 | 10.32 | <6.4 | <3.2 | 3.61 | 306.05 | 346.39 | 13.02 | 2147.37 | 16.02 | <2.56 | 79.31 | 52.84 | 2504.06 |
| **Mean** | 21.58 | 15.33 | 23.87 | 88.29 | 1.05 | 743.11 | 1.32 | 71.45 | 15.53 | 2.79 | 5.48 | 664.31 | 1064.89 | 50.95 | 2949.10 | 46.81 | 7.00 | 249.70 | 149.87 | 2073.92 |
| **SD** | 12.77 | 15.28 | 33.54 | 82.57 | 0.38 | 1275.98 | 1.37 | 185.55 | 9.09 | 0.67 | 5.49 | 698.85 | 1258.67 | 43.11 | 1112.64 | 45.19 | 4.80 | 226.80 | 210.47 | 1803.37 |
| **Median** | 19.43 | 8.00 | 10.54 | 66.91 | 1.28 | 57.86 | 0.64 | 10.43 | 13.61 | 3.20 | 3.60 | 492.86 | 479.75 | 34.24 | 2752.26 | 26.09 | 4.98 | 151.71 | 72.06 | 1175.22 |
| **IQR** | 10.07 | 16.43 | 18.80 | 50.22 | 0.46 | 832.13 | 0.78 | 8.39 | 10.85 | 0.60 | 8.13 | 308.44 | 541.03 | 27.72 | 782.41 | 42.83 | 7.56 | 273.96 | 122.16 | 1554.81 |
| **% Elevated** | 10.0 | 0.0 | 20.0 | 70.0 | 0.0 | 100.0 | 30.0 | 100.0 | 0.0 | 0.0 | 0.0 | 100.0 | 90.0 | 70.0 | 10.0 | 30.0 | 40.0 | 90.0 | 80.0 | 100.0 |
| **P Value^** | 0.035 | 0.27 | 0.067 | 0.19 | 0.38 | 0.093 | 0.091 | 0.20 | 0.12 | 0.15 | 0.43 | 0.15 | 0.20 | 0.41 | 0.215 | 0.38 | 0.045 | 0.32 | 0.36 | 0.23 |
| ^δ^ Concentration values highlighted in gray are considered elevated above the normal control mean + 2xSD; unit is pg/ml unless otherwise noted | | | | | | | | | | | | | | | |  |  |  |  |  |
| * Recipient deceased by Day 10  ^T-test was used to compare Means of Track 2 and Track 3  SD, Standard Deviation of the Mean; IQR, Interquartile Range  Median, IQR, Mean and SD were calculated using the lowest value detected for any value listed as (<). | | | | | | | | | |  |  |  |  |  |  |  |  |  |  |  |
