# Supplementary material for: Elevated cytokines and chemokines in peripheral blood of patients with SARS-CoV-2 pneumonia treated with high-titer convalescent plasma
Source: PLoS Pathog. 2021 Oct 29;17(10):e1010025. doi: 10.1371/journal.ppat.1010025 (PMC8580259; doi:10.1371/journal.ppat.1010025)
Supplement: S10 Table — (DOCX) [file ppat.1010025.s011.docx]

**S10 Table. Individual Recipient Plasma Ig Isotype Concentrations Over Time**

|  |  | **IgM** |  |  | **IgG1** |  |  | **IgG2** |  |  | **IgG3** |  |  | **IgG4** |  |  | **IgA** |  |
| --- | --- | --- | --- | --- | --- | --- | --- | --- | --- | --- | --- | --- | --- | --- | --- | --- | --- | --- |
| **Recipient** | **Pre-Infusion** | **Day 3** | **Day 10** | **Pre-Infusion** | **Day 3** | **Day 10** | **Pre-Infusion** | **Day 3** | **Day 10** | **Pre-Infusion** | **Day 3** | **Day 10** | **Pre-Infusion** | **Day 3** | **Day 10** | **Pre-Infusion** | **Day 3** | **Day 10** |
| **TRACK 2** | | | | | | | | | | | | | | | | | | |
| REC01 | 1526.64 | 1021.93 | 2043.42 | 1121.67 | 1801.07 | 4467.36 | 1943.82 | 1344.10 | 2661.26 | 138.83 | 138.15 | 364.37 | 592.81 | 476.70 | 4011.37 | 1132.25 | 920.50 | 1585.84 |
| REC03 | 1068.54 | 773.43 | * | 2375.09 | 3054.17 | * | 2017.37 | 1432.03 | * | 180.19 | 167.21 | * | 183.60 | 149.43 | * | 892.29 | 683.32 | * |
| REC05 | 324.92 | 376.48 | 568.75 | 1740.95 | 2614.30 | 6585.11 | 1257.45 | 746.03 | 594.30 | 224.75 | 225.14 | 304.85 | 716.91 | 613.58 | 4953.83 | 879.62 | 911.67 | 1544.76 |
| REC06 | 272.60 | 221.74 | 657.67 | 1443.55 | 1929.41 | 6228.04 | 881.60 | 969.82 | 2039.93 | 190.92 | 240.67 | 2619.55 | 61.21 | 109.01 | 195.95 | 1371.21 | 1178.44 | 2780.93 |
| REC07 | 474.17 | 410.80 | 503.19 | 1650.70 | 2064.34 | 2278.42 | 2182.91 | 1607.83 | 2566.62 | 113.87 | 141.62 | 99.81 | 5284.39 | 4516.99 | 6080.95 | 1521.67 | 1138.96 | 1565.84 |
| REC08 | 697.06 | 350.24 | 510.14 | 2539.29 | 3183.28 | 3897.74 | 1201.34 | 1414.59 | 1046.54 | 472.82 | 358.88 | 528.31 | 5284.21 | 80.95 | 44.68 | 1461.75 | 807.30 | 937.70 |
| REC09 | 951.11 | 727.46 | 1459.91 | 2492.72 | 2996.38 | 4731.91 | 976.00 | 607.38 | 482.24 | 453.74 | 574.95 | 2449.67 | 377.65 | 322.91 | 320.34 | 1173.14 | 1127.41 | 1415.38 |
| REC10 | 458.01 | 321.08 | 289.03 | 1025.81 | 1095.08 | 1552.15 | 1160.84 | 627.42 | 391.35 | 79.48 | 63.04 | 172.55 | 52.26 | 69.54 | 122.31 | 105.50 | 127.47 | 202.78 |
| REC11 | 214.32 | 96.45 | 304.91 | 1740.58 | 1271.23 | 4041.37 | 1998.63 | 1113.82 | 3223.35 | 166.11 | 103.64 | 552.89 | 74.27 | 53.88 | 202.76 | 1159.25 | 629.78 | 2028.92 |
| REC13 | 456.40 | 579.98 | 998.65 | 2950.98 | 3382.71 | 5167.20 | 1592.47 | 935.23 | 1688.71 | 780.56 | 1931.70 | 3068.62 | 49.48 | 52.13 | 78.41 | 803.19 | 884.45 | 1242.49 |
| REC15 | 592.03 | 702.43 | 793.36 | 2586.77 | 3607.79 | 3962.43 | 2293.70 | 1738.94 | 1494.96 | 698.36 | 709.17 | 645.68 | 369.80 | 368.13 | 264.87 | 1471.71 | 1582.04 | 1378.63 |
| REC16 | 958.68 | 652.66 | 736.52 | 3394.79 | 2651.22 | 3658.68 | 2127.72 | 1238.07 | 1927.88 | 2221.34 | 800.77 | 2161.96 | 168.11 | 159.77 | 189.39 | 1420.08 | 1058.32 | 1245.96 |
| REC17 | 486.26 | 324.10 | 865.33 | 2072.13 | 2032.82 | 3137.97 | 2660.68 | 953.04 | 1365.22 | 1317.07 | 585.58 | 3037.08 | 2934.60 | 895.87 | 920.35 | 1464.65 | 975.37 | 1101.34 |
| REC22 | 1240.08 | 1377.92 | 871.99 | 1501.79 | 4377.47 | 3124.37 | 805.10 | 745.93 | 134.93 | 132.75 | 336.42 | 576.71 | 28.99 | 40.73 | 45.81 | 421.39 | 771.82 | 588.01 |
| REC24 | 500.87 | 476.71 | 854.02 | 1508.58 | 2538.12 | 4772.86 | 1145.17 | 827.37 | 1194.48 | 204.00 | 300.00 | 772.81 | 4755.83 | 6012.85 | 8590.91 | 550.00 | 616.35 | 874.43 |
| REC25 | 498.05 | 614.29 | 959.45 | 1443.55 | 3251.71 | 4069.56 | 2660.79 | 1965.62 | 3210.07 | 365.93 | 700.87 | 1155.22 | 558.19 | 601.52 | 537.19 | 1193.09 | 1268.80 | 1313.32 |
| REC27 | 774.08 | 1025.59 | 1183.18 | 771.25 | 2149.92 | 2681.08 | 1311.72 | 1440.71 | 1981.01 | 299.54 | 750.13 | 988.56 | 78.51 | 162.98 | 115.81 | 1102.55 | 1579.57 | 1471.19 |
| REC33 | 633.35 | 652.33 | 2013.06 | 1035.44 | 2046.41 | 4257.66 | 672.29 | 1379.44 | 1886.94 | 431.83 | 872.39 | 2789.60 | 2780.87 | 5986.73 | 9193.77 | 499.56 | 700.87 | 871.21 |
| REC35 | 490.97 | 660.60 | 1077.26 | 2020.12 | 3432.45 | 6231.04 | 2458.16 | 2398.19 | 4841.17 | 2054.35 | 2952.21 | 4081.00 | 569.11 | 587.70 | 4680.80 | 1885.00 | 1933.61 | 3362.65 |
| REC37 | 250.21 | 480.72 | 983.90 | 1274.84 | 2739.69 | 3180.85 | 557.28 | 413.28 | 228.43 | 401.76 | 1986.75 | 3037.80 | 20.38 | 162.34 | 90.03 | 899.62 | 1210.86 | 1686.96 |
| REC38 | 826.84 | 879.72 | 1307.02 | 904.03 | 1541.28 | 2184.37 | 1703.66 | 1291.13 | 1887.58 | 327.34 | 530.21 | 731.87 | 11.44 | 69.52 | 45.55 | 553.67 | 706.75 | 1088.31 |
| REC39 | 899.02 | 1133.53 | 1222.23 | 734.60 | 2351.06 | 3034.76 | 1142.29 | 1060.39 | 1347.41 | 260.37 | 601.65 | 850.00 | 300.04 | 649.57 | 751.63 | 453.18 | 837.11 | 852.34 |
| REC40 | 29.46 | 102.44 | 63.78 | 467.84 | 633.39 | 471.80 | 7485.44 | 245.53 | <41.15 | 6.53 | 28.51 | 14.40 | 1.28 | 15.36 | 5.34 | 55.41 | 143.76 | 85.53 |
| **Mean** | 635.81 | 607.07 | 921.22 | 1686.83 | 2467.19 | 3805.31 | 1836.37 | 1151.99 | 1798.30 | 500.98 | 656.51 | 1409.24 | 1098.00 | 963.40 | 1883.73 | 976.95 | 947.59 | 1328.39 |
| **SD** | 355.04 | 328.10 | 497.52 | 763.37 | 892.29 | 1515.92 | 1387.54 | 505.30 | 1126.12 | 589.09 | 716.05 | 1232.84 | 1771.44 | 1833.56 | 2942.47 | 485.37 | 419.35 | 735.01 |
| **Median** | 500.87 | 614.29 | 868.66 | 1508.58 | 2538.12 | 3930.08 | 1592.47 | 1113.82 | 1591.84 | 299.54 | 530.21 | 811.41 | 300.04 | 162.98 | 233.81 | 1102.55 | 911.67 | 1279.64 |
| **IQR** | 405.72 | 387.09 | 565.72 | 1145.05 | 1137.61 | 1608.61 | 1011.59 | 636.61 | 1317.84 | 290.13 | 533.48 | 2042.62 | 598.12 | 532.31 | 3142.14 | 843.81 | 454.89 | 670.32 |
| **% Elevated** | 4.3 | 0.0 | 13.6 | 0.0 | 0 | 22.7 | 4.3 | 0.0 | 13.6 | 13.0 | 13.0 | 45.4 | 26.1 | 17.4 | 36.4 | 4.3 | 4.3 | 13.6 |
| **Control Mean + 2xSD** |  | 1392.28 |  |  | 4769.89 |  |  | 2713.59 |  |  | 978.25 |  |  | 697.03 |  |  | 1878.46 |  |
|  |  |  |  |  |  |  |  |  |  |  |  |  |  |  |  |  |  |  |
| **TRACK 3** |  |  |  |  |  |  |  |  |  |  |  |  |  |  |  |  |  |  |
| REC02 | 279.60 | 239.34 | 389.44 | 1342.69 | 1533.42 | 2259.71 | 1163.83 | 1015.38 | 1169.38 | 509.40 | 463.44 | 1171.67 | 548.04 | 398.24 | 488.48 | 686.36 | 636.55 | 831.74 |
| REC04 | 429.17 | 349.14 | 312.28 | 3379.53 | 3014.77 | 8232.79 | 2109.73 | 1122.71 | 3685.76 | 2119.72 | 742.13 | 3378.10 | 360.15 | 239.60 | 2810.92 | 1774.22 | 1183.57 | 2323.40 |
| REC12 | 322.38 | 710.65 | * | 1887.02 | 4363.01 | * | 1759.01 | 1220.32 | * | 301.96 | 2204.29 | * | 113.97 | 6955.33 | * | 734.23 | 1598.57 | * |
| REC14 | 1100.43 | 953.38 | 860.73 | 2921.14 | 3679.36 | 4001.71 | 1923.54 | 1405.70 | 1296.40 | 374.07 | 398.81 | 390.16 | 35.13 | 62.29 | 20.66 | 3076.73 | 2479.88 | 2143.19 |
| REC18 | 632.78 | 411.90 | 179.98 | 1870.00 | 1470.10 | 1935.83 | 1126.23 | 331.62 | 379.37 | 121.38 | 82.28 | 124.69 | 132.44 | 150.39 | 134.31 | 1672.67 | 1123.11 | 1245.17 |
| REC19 | 595.85 | 747.49 | 407.51 | 930.77 | 2183.93 | 1572.92 | 1443.58 | 1423.37 | 252.67 | 374.67 | 833.94 | 704.21 | 1164.21 | 4341.35 | 717.30 | 917.57 | 1473.77 | 1011.75 |
| REC21 | 496.00 | 506.31 | 338.27 | 1805.63 | 2835.88 | 2327.28 | 3593.54 | 3137.44 | 2119.40 | 381.85 | 452.35 | 384.37 | 12.55 | 33.72 | 8.04 | 1996.53 | 2080.63 | 1264.91 |
| REC23 | 1810.14 | 2085.05 | 2821.53 | 1231.34 | 2161.16 | 1939.01 | 1332.02 | 925.83 | 462.21 | 62.69 | 114.83 | 76.30 | 31.37 | 43.01 | 26.48 | 606.79 | 789.08 | 703.14 |
| REC26 | 228.56 | 375.90 | 601.47 | 1130.43 | 2456.65 | 3519.64 | 767.72 | 350.04 | 2790.10 | 134.86 | 326.44 | 184.64 | 188.21 | 314.96 | 214.34 | 261.73 | 460.94 | 617.71 |
| REC29 | 764.66 | 844.00 | 684.00 | 840.73 | 2170.81 | 2131.10 | 1682.12 | 1721.46 | 1663.90 | 258.46 | 610.43 | 545.38 | 1005.87 | 4728.72 | 2010.69 | 493.33 | 729.92 | 568.51 |
| REC34 | 191.47 | 472.37 | * | 598.72 | 1215.20 | * | <41.15 | 100.52 | * | 14.99 | 64.43 | * | 0.51 | 15.56 | * | 36.17 | 156.41 | * |
| REC36 | 971.85 | 597.68 | 521.61 | 5565.71 | 5223.11 | 3384.17 | 2714.18 | 1564.09 | 1379.78 | 105.06 | 179.93 | 93.85 | 310.65 | 208.54 | 141.29 | 2070.08 | 1418.19 | 928.80 |
| **Mean** | 651.91 | 691.10 | 711.68 | 1958.64 | 2692.29 | 3130.42 | 1783.23 | 1193.21 | 1519.90 | 396.59 | 539.44 | 705.34 | 325.26 | 1457.64 | 657.25 | 1193.87 | 1177.55 | 1163.83 |
| **SD** | 464.52 | 488.77 | 767.45 | 1405.92 | 1212.88 | 1963.08 | 802.90 | 800.61 | 1100.62 | 563.87 | 582.10 | 998.16 | 392.26 | 2421.05 | 969.69 | 910.19 | 676.74 | 612.29 |
| **Median** | 545.92 | 552.00 | 464.56 | 1574.16 | 2320.29 | 2293.49 | 1682.12 | 1171.52 | 1338.09 | 280.21 | 425.58 | 387.27 | 160.33 | 224.07 | 177.82 | 825.90 | 1153.34 | 970.27 |
| **IQR** | 504.78 | 368.72 | 312.31 | 1065.04 | 1176.69 | 1498.74 | 768.71 | 676.67 | 1366.52 | 259.16 | 479.70 | 524.82 | 372.93 | 1326.54 | 606.66 | 1251.37 | 798.39 | 524.68 |
| **% Elevated** | 8.3 | 8.3 | 10.0 | 8.3 | 0 | 10.0 | 16.7 | 8.3 | 20.0 | 8.3 | 8.3 | 20.0 | 16.7 | 25.0 | 30.0 | 25.0 | 16.7 | 10.0 |
| **P Value^** | 0.46 | 0.30 | 0.22 | 0.27 | 0.29 | 0.18 | 0.44 | 0.44 | 0.26 | 0.31 | 0.30 | 0.051 | 0.028 | 0.27 | 0.045 | 0.23 | 0.15 | 0.26 |
| ^δ^ Concentration values (ug/ml) in highlighted in gray are considered elevated above the normal control mean + 2xSD | | | | | | | | | | | |  |  |  |  |  |  |  |
| * Recipient deceased by Day 10  ^T-test was used to compare Means of Track 2 and Track 3  SD, Standard Deviation of the Mean; IQR, Interquartile Range | | | | | | |  |  |  |  |  |  |  |  |  |  |  |  |
