# Supplementary material for: Elevated cytokines and chemokines in peripheral blood of patients with SARS-CoV-2 pneumonia treated with high-titer convalescent plasma
Source: PLoS Pathog. 2021 Oct 29;17(10):e1010025. doi: 10.1371/journal.ppat.1010025 (PMC8580259; doi:10.1371/journal.ppat.1010025)
Supplement: Attachment — Submitted filename: PLOS Pathogens_Response to Reviewers Comments_FINAL.docx [file ppat.1010025.s012.docx]

CPT Manuscript PLOS Pathogens reviewer comments

**Part II – Major Issues: Key Experiments Required for Acceptance**
Please use this section to detail the key new experiments or modifications of existing experiments that should be absolutely required to validate study conclusions.

Generally, there should be no more than 3 such required experiments or major modifications for a "Major Revision" recommendation. If more than 3 experiments are necessary to validate the study conclusions, then you are encouraged to recommend "Reject".

Reviewer #1: 1. It will be much more clearly and convinced if the tables characterizing the patients were divided into two groups: track2 and track 3, and calculated the P value. Tables IV, V, VI, VIII, and IX were reformatted to split the patient cohort by track. P values of the comparison of Means between Track 2 and Track 3 are presented in each Table.
2. For the two groups (Track 2 and Track 3), the infusion titers of neutralizing anti-SARS-CoV-2 antibodies were the same or not? There was no significant difference in the percentage of patients in either track receiving different infusion titers; 26% of patients in Track 2 and 25% of patients in Track 3 received titers >10,000, 69.5% of patients in Track 2 and 66.6% of patients in Track 3 received titers of 1,000-10,000, 4.3% of Track 2 patients and 8.3% of Track 3 patients received titers of 500-1,000. In our previous publication no difference in clinical endpoints was detected within the ranges of donor IgG antiviral titers used (lines 239-244).
3. Other treatments were same or not for the two groups? Especially anti-viral drug, corticoids? How about the rates of comorbidity for the two groups? This data is now presented in Table III. There were no statistical differences between tracks in regards to comorbidities. In regards to concomitant treatment, the use of corticosteroids and tociluzumab was greater in the Track 3 patients. This would be expected given the more severe clinical condition of patients within that cohort. The effect this might have on our data analysis is discussed in the revised text (lines 563-570).
4. “for either a planned fresh infusion of 500 mL or for cryopreservation in aliquots of 200 mL”, this might make differences. The assays performed in this study were done on frozen aliquots of all donor and patient samples (line 237-239). In addition, in the original study, there were no differences in measured endpoints for each track when using either fresh or frozen plasma (line 166-167) and therefore we did not analyze differences in cytokine composition in this fashion.
5. For better comparison, if the detection data of track2 and 3 were put in the same figures. Due to the quantity of data presented, it would not be feasible to combine the data for Track 2 and Track 3 in the same figures while still comparing changes over time. In order to keep the data presented in a clear fashion, we have decided to keep the figures separated by Tracks.
6. The number of patients were limited to draw the conclusion. We recognize that the number of patients in this arm of the study was limited. This was addressed as a limitation in the discussion section of the original manuscript. (line 552) In addition, we have revised our language in several areas to reflect that the conclusions drawn from our study would need to be confirmed with a more robust study.

Reviewer #2: 1. Limitation section should further discuss the lack of a control group with corresponding cytokine/chemokine and Ig levels that limits establishing any causality between CPT and change in the measured levels. The findings such as elevated IL-6 and CRP may be an expected findings of severe Covid-19 and no inference can be made regarding the effect of CPT or how the findings can be utilized as biomarkers in using CPT. To address this concern, we analyzed cryopreserved plasma samples from hospitalized COVID-19 patients treated at Hackensack University Medical Center. The Biorepository provided sequential samples from 8 patients in Track 2 and 10 patients in Track 3 who did not receive convalescent plasma. Samples were selected based on proximity to collection time points in CPT cohort (day 10 post-symptom onset for Track 2 and day 15 for Track 3, followed by samples collected approximately 10 days later corresponding to the day 10 post-infusion timepoint). The data, presented in Figure 7, examined the change in cytokine concentration (delta) over the 10-day study period. T test analysis revealed statistically significant deltas in several cytokines between those patients that received CPT compared to those who did not (nonCPT). This newly added data is discussed in the text (line 150-159; 407-430; 534-550). The effect size of the logistic regression finding was small despite statistical significance limiting clinical meaningfulness of the finding. The fact that the effect size was small has been added to the discussion (line 526-527). Additionally, some of the relevant clinical variables that may be confounders were not taken into account (such as age, sex, BMI, concomitant medications) and small sample size may have limited further analysis. While the small sample size limited our ability to assess confounders, Table III now presents statistical analysis comparing recipient characteristics in Track 2 and Track 3 patients. There were no statistical differences between tracks in regards to age, sex, BMI and comorbidities. In regards to concomitant treatment, the use of corticosteroids and tociluzumab was greater in the Track 3 patients. While this is to be expected given the more severe clinical condition of patients within that cohort, it is possible that these concomitant treatments could impact cytokine production. This is addressed in the limitations section of the discussion (line 563-570) To gain more insight into the association between CPT and the change in cytokine/chemokine levels in this single arm study, it may be interesting to analyze by CP titers or baseline serostatus of CP recipients if sample size allows. Our study was designed to evaluate “high-titer CP” and was not powered to evaluate the optimal donor antiviral titer level (line 475-477).

2. The presentation of the data may be improved by revising the formats of the tables. Alternative to presenting raw individual data, it may be helpful to present the descriptive findings as median (IQR) or mean (SD) depending on the distribution for continuous variables and freq (%) for categorical variables. For Tables IV, V, VI, and IX, we revised as suggested presenting the descriptive statistics including Range, Median, IQR, Mean, and Standard deviation as well as p values for the comparison of the Means of the Track 2 and Track 3 cohort. Tables with raw individual data have been added to the supplemental data tables and now include Median and IQR as well (See supplemental Tables, VII, VIII, IX, and X).

3. In the 2nd and 3rd paragraph of the discussion section, authors make speculative statements regarding possible transfer of inflammatory cytokine/chemokines from CP unit to the recipients and the effect of IgG4 in CP unit that are not sufficiently supported by the data presented. Causality is inferred from their observations of cytokine/chemokine levels in small number of CP units and recipients’ plasma and suggest possible transfer of the cytokines from CP unit to recipient. The text was changed to emphasize that these inferences are purely speculative and further research would need to be conducted to support causality. (line 472-473) Additionally, this hypothesis was not clearly prefaced in introduction or methods. It may help to clarify in the introduction section if the authors are suggesting that CPT had an immunomodulatory effect or caused the elevation of cytokine/chemokines associated with cytokine storm. Statement in line 415, 416 “exclusion of donor plasma with multiple cytokine elevations might be appropriate as an extra precaution” seem to be relatively strong without sufficient supporting data. The statement in line 415-416 (now line 456-458) was rephrased to reflect that a more robust study would need to be completed to confirm this hypothesis.

**Part III – Minor Issues: Editorial and Data Presentation Modifications**
Please use this section for editorial suggestions as well as relatively minor modifications of existing data that would enhance clarity.

**Reviewer #1:** Page 7, line 44, “A phase IIb clinical trial”, Page 11, line 116, “phase IIa clinical trial”, IIa or IIb? This was a phase IIa clinical trial. The text on line 44 was corrected.

**Reviewer #2**:

1. Some of the results are presented in the introduction and discussion section. For example, Page 6, lines 105-108 may fit better in the results section. Some of the text describing the findings of our study were removed from the introduction as suggested.
2. Consider reporting the ‘n’ in the figures when available, presence of any missing samples, and if blood samples were drawn in all participants or only those who remained hospitalized at day 10. N values were added to figure legends. Samples were taken from all living patients on day 10, regardless of hospitalization status (line 169-170). Patients who succumbed prior to day 10 sample collection are indicated by * in appropriate Tables and Figures.
3. Page 5, Line 91, 92: Consider providing details on how the non-CPT treated subjects in the original study were identified – whether they were from a randomized prospective study or were retrospectively identified control group. Non-CPT treated patients were treated for COVID-19 within our hospital network system. Data was collected retrospectively from the electronic health records. Patients with characteristics closest to the patients in our two treatment cohorts were selected for analysis. (line 92-93)
4. Page 5, Line 92: Can state P=0.036 instead of P<0.036? The text has been corrected as indicated.
5. Page 6, line 110-112: It is not clear what the authors mean that identifying biomarkers will improve outcome in the pre- and post-vaccination period. This text was removed as the first draft was written when all of the COVID-19 vaccines in development had yet to be approved either fully or for emergency use.
6. page 10, line 184. It is not clear why there is a range, 12-16, of healthy adult donors. Additionally, would clarify if the healthy donors were screened for SARS-CoV-2 and were negative. The n value varies for each analyte depending upon how many wells were available for controls on the 96-well plate for each assay (see Supplemental Table V). Healthy donor samples were selected from cryopreserved plasma collected prior to 2019 to ensure the controls were SARS-CoV-2 negative (see added text, line 198-200).
7. page 10, line 185-186. What is the criterion used for elevation of correlates (ie. values greater than 2 SD from the mean of the healthy controls) based on? Is it a standard definition? With normally distributed data, 2SD from the mean represents the 95% confidence interval.
8. page 10, line 191. It is not clear how progression to positive pressure mechanical ventilation was measured. If it is delineated in the original paper, would include the reference. The decision to put Track 2 patients on mechanical ventilation was a clinical decision from the intensive care team based on multiple standard parameters that pointed to poor respiratory function (eg., oxygen levels, respiratory muscle fatigue, excessive work of breathing, etc). The decision to place a patient on mechanical ventilation was made independently from the research study team in order to minimize bias. (see added text, line 207-211)
9. page 10, line 203: Would clarify if mean (SD) and t test were used based on normal distribution of the data. Consider including statistical program used for completeness. The patient data was not assessed for normal distribution. The statistical program R was used for analysis. This was added to the Statistical Analysis section of the Materials and Methods (line 222-223).
10. page 12, line 208-213: Details regarding the donors in the result section seem repetitive as that was presented in the methods section. All details regarding donor screening and selection criteria were moved to the Materials and Methods section.
11. page 12, line 212. Is “infectious disease markers” a standardized test for pathogens that can be transmitted by transfusion? Yes, this is a universal panel that is run on all transfusion products.
12. page 13, line 233. It is not clear which font is marked in red. Is it referring to the values highlighted in grey in table 2A? This was corrected to highlighted in gray.
13. Recommend reformatting table 3, patient characteristics. Continuous variables can be presented as median (IQR) and categorical variables as freq (%). Consider presenting the number of days from admission to transfusion which can give an insight on how early in admission the patients were transfused. Table III was reformatted as suggested and now includes statistical comparisons between recipient characteristics in the Track 2 compared to Track 3 cohorts. Continuous variables were analyzed using T test, categorical variables were analyzed using Fisher’s exact test. Data on the number of days from admission to transfusion was not readily available.
14. Race variable is categorized as Caucasian, African American, Hispanic and Asian. Hispanic vs non Hispanic should be included in a ethnicity variable. Consider making Race/Ethnicity variable and categorize as non-hispanic white, non-hispanic black, Hispanic etc. After considering the suggested change, we decided to keep the race/ethnicity data as presented in order to maintain consistency with how this data was depicted in the publication of our original clinical study (Reference #21, Donato et al, JCI 2021)

5. page 14, line 251. Can include measure of dispersion (IQR) in addition to median. Text was changed to reflect Median (IQR) as suggested and now corresponds with presentation in Table III. This revision can be found on line 272-275.
16. page 14, lines 253-254. Repetitive to have definitions of track 2 and 3 reported again. Definitions were deleted to avoid repetition
17. page 22, line 433, 434. Authors state, “in regard to the anti-SARS-CoV-2 neutralizing IgG titers, we initially hypothesized that selecting CP donors with high titers would optimize the benefit to the recipients”. It is not clear if this dose response was examined and if this initial hypothesis was supported by the author’s data or if this is a general statement. May consider editing the sentence slightly to clarify. Our study was not powered or designed to evaluate the optimal donor antiviral titer level, or the optimal dose of IgM and IgG to be infused. The above statement was edited to clarify this. It now reads, “In regard to the anti-SARS-CoV-2 neutralizing IgG titers, we initially postulated that selecting convalescent plasma donors with high titers would optimize the benefit to the recipients, although our study was not designed to evaluate optimal donor neutralizing antibody titers.” (line 475-477)
